# Natural products as HMG-CoA reductase inhibitors (statins) for the management of non-communicable diseases

**DOI:** 10.1007/s10787-025-02069-2

**Published:** 2025-12-18

**Authors:** Radwa N. Muhammad, Nora M. Aborehab, Shaza H. Aly, Noha N. Nasr, Merhan O. Hindam, Aya M. Mustafa, Safaa A. Faheem, Mariam H. Fawzy, Mohammed E. Abo-El Fetoh, Ahmed M. El-Dessouki, Mostafa A. Rabie, Riham A. El-Shiekh, Mahmoud E. Youssef

**Affiliations:** 1 Department of Pharmacology and Toxicology, Faculty of Pharmacy, Cairo University, Cairo, 11562 Egypt; 2 Department of Biochemistry, Faculty of Pharmacy, Ahram Canadian University, Giza, 12566 Egypt; 3 Department of Pharmacognosy, Faculty of Pharmacy, Badr University in Cairo (BUC), Cairo, 11829 Egypt; 4 Therapeutic Chemistry Department, Pharmaceutical and Drug Industries Research Institute, National Research Center, Dokki, Giza, Egypt; 5 Department of Pharmacology and Toxicology, Faculty of Pharmacy, Egyptian Russian University, Cairo, Egypt; 6 Pharmacology and Toxicology Department, Faculty of Pharmacy, Ahram Canadian University, 6th of October City, 12566 Giza Egypt; 7 Department of Pharmacognosy, Faculty of Pharmacy, Cairo University, Kasr El-Aini Street, Cairo, 11562 Egypt; 8 Department of Pharmacology and Biochemistry, Faculty of Pharmacy, Delta University for Science and Technology, Gamasa, 11152 Egypt

**Keywords:** Statins, Noncommunicable diseases, CVD, CNS, Cancer, Autoimmune, Kidney, Gastrointestinal, Eye disorders, Natural products

## Abstract

Non-communicable diseases, such as diabetes, cancer, as well as cardiovascular, metabolic, and central nervous system disorders stand for significant global health burden. Statins, as HMG-CoA reductase inhibitors, have emerged as a basis in the management of hypercholesterolemia and the prevention of cardiovascular disorders. In this review, we summarize the role of statins in a variety of pathologies, ranging from their well-recognized effects in cardiovascular disorders, to the newly revealed favorable effects in cancer, eye, autoimmune, kidney, gastrointestinal, bone, and autoimmune disorders. Additionally, the current review explores insights into the potential role of natural products as HMG-CoA reductase inhibitors. Future research should focus on the synergistic effects of natural therapies with synthetic statins to improve health outcomes in populations burdened by non-communicable diseases.

## Introduction

Statins, also known as 3-hydroxy-3-methylglutaryl-coenzyme (HMG-CoA) reductase inhibitors, are the first-line pharmacological therapy to lower serum low density lipoprotein (LDL) cholesterol levels (Smith et al. 2009). Over the last two decades, the use of HMG-CoA reductase inhibitors, or statins, has increased in frequency and intensity for secondary cardiovascular (CV) event reduction, as well as primary event prevention in high-risk populations (Mahdavi et al. 2020). Non-communicable diseases (NCDs) are the main cause of death and pose an increasing danger to global health. NCDs accounted for 71% (41 million) of the total death cases (57 million) that occurred globally. Among the NCDs, CV diseases (CVDs) (31%), malignancies (16%), injuries (9%), chronic respiratory illnesses (7%), and diabetes (3%) are the leading contributors to global fatalities (Wong et al. 2020). The increasing prevalence of behavioral and metabolic risk factors (such as overnutrition, physical inactivity, obesity, hypertension, alcohol use, and tobacco use) leads to NCDs bearing the majority of the disease burden. The excessive inflammatory response and oxidative stress serve as both the antecedent and manifestation of NCDs (Camps and García-Heredia 2014). The currently existing pharmacological drugs used to treat NCDs are with side effects, necessitating the search for novel treatments with greater efficacy and fewer adverse effects. Researchers are currently very interested in the use of nutraceuticals as adjuvant therapies due to their possible safety, nutritive, and therapeutic effects (Nasri et al. 2014). Several studies reported the potential herbal medicines as HMG-CoA reductase inhibitors (Mahdavi et al. 2020). Hence, we thought it would be wise to document the efficiency of medicinal plants to inhibit the HMG-CoA reductase enzyme. In light of the promising evidence regarding the impact of medicinal plants on HMG-CoA reductase activity, we believe it is prudent to document the efficacy of these natural substances in inhibiting this critical enzyme. Such documentation will contribute to the potential integration of herbal remedies into conventional therapeutic strategies for managing NCDs and preventing related health conditions.

### HMG-CoA reductase inhibitors and cardiovascular disorders

#### Overview

HMG-CoA reductase inhibitors have become the cornerstone of therapy for managing CVDs. They were originally conceived as hypolipidemic agents; however, statins have garnered continued interest due to their anti-inflammatory, antioxidant, and endothelial-stabilizing effects (Wazir et al. 2023). Statins are effective in treating various CV conditions such as heart failure (HF), arrhythmias, myocardial infarction (MI), angina, and atherosclerosis. In fact, CVDs are the leading cause of death across the globe, associated with substantial morbidity and huge healthcare spends (Chong et al. 2024). Dyslipidemia, in particular, raised levels of LDL stands as one of the most significant risk factors in developing atherosclerotic CVD (ASCVD) (Abera et al. 2024). The statins, through inhibition of the cellular enzyme HMG-CoA reductase, favorably lower the LDL levels and hence risk for major CV events. Their actions extend far beyond lowering cholesterol levels and include extensive pathways involved in inflammatory response, oxidative stress, and endothelial dysfunction, as well as thrombus formation. Thus, statins become indispensable in the management of all ranges of CVDs from stable angina through acute MI and on to HF **(**Table 1**).**

**Table 1:** Role of statins in CVD

Condition	Molecular Mechanisms	Outcomes
HF	Inhibition of Rho and Rac GTPases, NADPH oxidase, enhancement of eNOS, reduction of proinflammatory cytokines (e.g., TNF-α, IL-6), inhibition of TGF-beta, and increased PPAR-γ coactivator (PGC)-1α for mitochondrial function	Improved cardiac function, reduced inflammation and fibrosis, enhanced myocardial perfusion
Arrhythmias	Reduction in ROS, upregulation of eNOS, stabilization of Na+, K+, and Ca2+ channels, inhibition of TGF-β/MMP, and autonomic balance restoration	Decreased arrhythmia burden, improved electrophysiology, and reduced atrial remodeling
MI	Activation of RISK pathway (Akt, ERK1/2, PKC), inhibition of mPTP opening, VEGF and HIF-1α upregulation for angiogenesis, and reduced thrombogenesis (e.g., NO bioavailability)	Reduced ischemic injury, enhanced perfusion, and decreased risk of future thrombotic events
Atherosclerosis	HMG-CoA reductase inhibition, prevention of LDL oxidation, enhancement of HDL-mediated cholesterol efflux, NF-κB inhibition, and stabilization of plaques through reduced MMP activity and macrophage infiltration	Slowed plaque progression, reduced risk of rupture, and decreased cardiovascular events

Akt, protein kinase B; eNOS, endothelial nitric oxide synthase; ERK, extracellular signal-regulated kinase; CVD, cardiovascular disease; GTP, guanine triphosphate; HDL, high density lipoprotein; HMG-CoA, hydroxymethylglutaryl-CoA; HIF, hypoxia-inducible factor; IL, interleukin, LDL, low density lipoprotein, MMP, matrix metalloproteinase, mPTP, mitochondrial permeability transition pore; NADPH, nicotinamide adenine dinucleotide phosphate; NF, nuclear factor; NO, nitric oxide; PKC, protein kinase C; PPAR, peroxisome proliferator–activated receptor; RISK, reperfusion injury salvage kinase; TGF, transforming growth factor; TNF, tumor necrosis factor.

#### Heart failure (HF)

HF arises from the progressive deterioration of myocardial function, particularly the heart’s contractile capacity, accompanied by maladaptive neurohormonal responses (Schwinger 2021). These changes contribute to the structural and functional remodeling of the left ventricles (LV). By acting on various cellular and molecular mechanisms involved in its pathogenesis, statins are able to modulate HF. One critical pathway in this context involves the inhibition of Rho and Rac GTPases. This is achieved by blocking their prenylation through the inhibition of isoprenoid synthesis, a process occurring in the cytoplasm of cells (Adam and Laufs 2013; Ahmadi et al. 2017). The primary signal transduction of oxidative stress and inflammation is mainly attributed to the activation of the oxidant enzyme NADPH oxidase by Rho and Rac GTPases. Reactive oxygen species (ROS) generated by this enzyme and its associated processes may establish a mechanistic link between statin therapy and the exacerbation of oxidative stress and endothelial injury—the hallmarks of HF pathophysiology (Hermida and Balligand 2014; Aimo et al. 2020).

Statins further enhance endothelium nitric oxide (NO) synthase (eNOS) expression and efficacy with increased production of NO. The latter could induce vasodilation, which would ultimately reduce the afterload and improve perfusion to the myocardium (Laufs 2003). In addition, statins reduce leukocytes migration and adhesion (Infante et al. 2011), decrease aggregation of platelets (An et al. 2019), and decrease proliferation of smooth muscle cells (Corpataux et al. 2005), leading to subsequent inhibition of inflammatory and fibrotic responses associated with HF. Moreover, the inhibition of transforming growth factor beta (TGF-β) signaling, which is an important profibrotic mechanism for activating fibroblasts and inducing extracellular matrix deposition, mediates the antifibrosis action (Rizvi et al. 2018). Therefore, statins improve LV compliance and overall cardiac function by lowering myocardial fibrosis.

Proinflammatory cytokines, such as tumor necrosis factor-alpha (TNF-α) and interleukin-6 (IL-6), are elevated in HF and contribute to disease progression. Statins decrease nuclear factor-kappa B (NF-κB) expression, which mediates the expression of proinflammatory cytokine in the context of myocardial and systemic inflammation, thus improving chronic inflammatory effects on the myocardium (Vogiatzi et al. 2017). Furthermore, statins improve mitochondrial function by increasing levels of peroxisome proliferator-activated receptor-gamma coactivator-1 alpha (PGC-1α), thus enhancing energy production and cardiomyocyte survival (Wang and Wong 2010). Based on these molecular factors, statins are believed to enhance therapeutic outcomes in patients with HF, particularly in the context of a pronounced inflammatory or oxidative stress component.

#### Arrhythmias

Atrial fibrillation (AF) and ventricular arrhythmias, often presenting with acute and severe manifestations, contribute significantly to the morbidity and mortality associated with CVDs. Statins are believed to ameliorate arrhythmia burden through modulation of structural, electrical, and autonomic remodeling (Williams et al. 2020). An important contributor to the development of arrhythmias is oxidative stress, which can lead to impaired ion channel functions and promotes atrial remodeling. Statins inhibit levels of ROS initiation through inhibiting NADPH oxidase and upregulating eNOS (Margaritis et al. 2017; Williams et al. 2020). Moreover, statins reestablish redox balance while stabilizing cardiac electrophysiology. This action has the effect of slowing the cardiac automaticity and reentry circuits that underlie arrhythmias (Oesterle and Liao 2019). Another important mechanism is that statins modulate ion channels. Statins stabilize the function of Na^+^, K^+^, and Ca^2+^ channels, which are often dysregulated in arrhythmias (Savelieva and Camm 2008; Williams et al. 2020). The normal action of these channels is, therefore, retained, thus maintaining the duration and conduction velocity for action potentials; this consequently decreases the chances for arrhythmias. Statins also inhibit atrial fibrosis, which constitutes an important substrate for AF, by repressing TGF-β signaling and matrix metalloproteinase (MMP) activity (Raschi et al. 2011; Li et al. 2021). Such effects reduce the deposit of extracellular matrix and also block structural remodeling.

Aside from oxidative stress and fibrosis, statins also modulate autonomic nervous function. These conditions ultimately mitigate the occurrence of triggered activity and the development of arrhythmias associated with autonomic dysfunction, specifically by reducing sympathetic overactivity and restoring balance to parasympathetic tone (Millar and Floras 2013). Their anti-inflammatory activities that encompass NF-κB inhibition would then reduce atrial remodeling through lowering C-reactive protein (CRP) and IL-6 levels (Parsamanesh et al. 2021). These molecular effects of statins could play a crucial role in preventing and treating atrial and ventricular arrhythmias.

#### Myocardial infarction (MI)

The primary cause of MI, ischemic injury and necrosis of the cardiomyocytes, relates to the rupture of atherosclerotic plaques and subsequent thrombosis (Młynarska et al. 2024). Extended-action statins employ a highly advantageous mechanism beyond their lipid-lowering effects for the management of both acute and chronic MI. This includes plaque stabilization achieved through reduced infiltration of inflammatory cells, a smaller lipid core size, and the suppression of MMP activity. This mechanism prevents rupture of plagues, which is the prime initiator of acute coronary events.

During ischemia–reperfusion injury, statins activate the reperfusion injury salvage kinases (RISK) pathway, including protein kinase B (PKB/Akt), extracellular signal-regulated kinase 1/2 (ERK1/2), and protein kinase C (PKC) (Hausenloy and Yellon 2004, 2007; Efthymiou et al. 2005; Bland et al. 2022). These kinases inhibit the opening of the mitochondrial permeability transition pore (mPTP), preventing apoptosis and allowing cell survival in cardiomyocytes (Jie et al. 2012; Penna et al. 2013). They also reduce thrombogenesis through decreased tissue factor expression, decrease platelet activation through raised NO bioavailability, and decrease thromboxane A2 levels, all of which led to reduced platelet aggregation and reduced thrombus formation.

Statins upregulate vascular endothelial growth factor (VEGF) and hypoxi-inducible factor 1 alpha (HIF-1α) in an ischemic myocardium, which leads to angiogenesis in such tissue. This improves collateral circulation enhances myocardial perfusion and recovery after MI (Yu et al. 2020). Besides, there is the stimulation of mobilization of endothelial progenitor cells (EPCs) to hasten endothelial repair and minimize the chances of future ischemic events (Sandhu et al. 2017).

#### Atherosclerosis

The leading cause of prevalent CVDs is a chronic inflammatory condition of the arterial wall known as atherosclerosis (Frąk et al. 2022). Statins target it at the molecular level as they inhibit HMG-CoA reductase, which then lowers LDL levels, causing an attenuation of inflammation. Lowering LDL decreases the lipid accumulation within plaques, thereby slowing down the rate of their progression and the chances of their rupture (Koushki et al. 2021). Table 2 summarizes different statin members potency and LDL reduction by intensity.

**Table 2:** Statins potency and LDL reduction by intensity

Statin	Typical dose range (mg/day)	Low-intensity dose (LDL reduction < 30%)	Moderate-intensity dose (LDL reduction 30% to < 50%)	High-intensity dose (LDL reduction ≥ 50%)
Atorvastatin	10–80 mg	–	10–20 mg	40–80 mg
Rosuvastatin	5–40 mg	–	5–10 mg	20–40 mg
Simvastatin	10–40 mg	10 mg	20–40 mg	–
Pravastatin	10–80 mg	10–20 mg	40–80 mg	–
Lovastatin	20–80 mg	20 mg	40–80 mg	–
Fluvastatin	20–80 mg	20–40 mg	40 mg (Twice/day)—XL 80 mg	–
Pitavastatin	1–4 mg	–	1–4 mg	–

LDL, low density lipoprotein.

Furthermore, statins inhibit the oxidation of LDL; it is critical for the formation of foam cells and also for the development of plaques. By increasing antioxidant defenses and lowering the levels of ROS, statins prevent oxidative modification of LDL particles (Hermida and Balligand 2014). Finally, statins have the potential for promoting reverse cholesterol transport by improving high density lipoprotein (HDL) functionality, thus leading to enhanced cholesterol efflux from macrophages in plaques into the liver for excretion from the body (Ouimet et al. 2019).

Inflammation has a starring role in atherosclerosis, and statins reduce it by blocking NF-κB activation. This decreases monocyte recruitment and cytokine production along with foam cell formation in plaques (Kouhpeikar et al. 2020). In addition, statins stabilize plaques through collagen induction, inhibition of MMP activity, and reduced macrophage infiltration (Libby and Aikawa 2003). All these effects increase the integrity of the fibrous cap and decrease the chances of rupture. Together, all these molecular actions create an essential space for statins as the most important medication for atherosclerosis management, significantly reducing morbidity and mortality from cardiovascular events.

### HMG-CoA reductase inhibitors and kidney diseases

#### Overview

Normal kidney function and the kidney’s reaction to metabolic insults depend on the adipose-renal axis, which connects adipose tissue to the kidney (Pereira et al. 2024). Kidney failure may result from increased levels of fatty acids, LDL cholesterol, and triglycerides (TG) in plasma, effects could be attributed to lipotoxicity, insulin resistance, and proinflammatory pathways (Gai et al. 2019). Indeed, lipotoxicity is the accumulation of excess cytosolic lipids in non-adipose cells that causes long-term cellular malfunction and damage (Opazo-Ríos et al. 2020). The primary causes of lipotoxicity are thought to include fatty deposits under the skin in the abdomen, raised plasma non-esterified fatty acid levels, compromised adipose tissue signaling, and ectopic lipid accumulation (Fang et al. 2024). Growing evidence suggests that lipotoxicity may contribute to a number of illnesses, including chronic kidney disease (CKD) (Weinberg 2006).

#### Chronic kidney disease (CKD)

CVD is recognized to be more common in people with CKD (Epstein and Campese 2005). Beyond the conventional risk factors linked to CVD, patients with CKD often have a variety of extra risk factors, such as electrolyte imbalances, proteinuria, inflammation, elevated oxidative stress, and changed NO levels (Uhlig et al. 2003). Hyperlipidemia is frequently linked to micro-albuminuria, particularly in individuals with diabetes and high blood pressure. Dyslipidemia, a common condition in hemodialysis users, increases the risk of CVD. Decreased HDL levels, elevated modified-LDL particles levels, and high levels of very low density lipoproteins (VLDL) and TGs are all present in these patients (Fellström et al. 2003).

Statins lower the production of liver cholesterol, and alter the metabolism of lipids through the inhibition of HMG-CoA reductase (Alhassani et al. 2021). Statins have a number of genuine advantages that exceed the hazards associated with the medication (Romani et al. 2019). Lipid-lowering medications may help CKD patients maintain their renal function, according to experimental and clinical data (Campese and Park 2007).

Kidney disease advances as a result of hyperlipidemia and hyperglycemia, which boost mesangial matrix synthesis and encourage inflammatory cells recruitment into the matrix (Weldegiorgis and Woodward 2022). Of note, LDL and oxidized-LDL stimulate the production of NF-κB and IL-6, which are two elements crucial to mesangial cells proliferative and inflammatory responses. NF-κB has been connected to glomerulonephritis-related inflammatory events (Epstein and Campese 2005). In the same context, simvastatin inhibited macrophage infiltration into glomeruli, mesangial matrix expansion, and mesangial cell proliferation in a rat model of glomerulonephritis (Yoshimura et al. 1999). Moreover, pravastatin reduced intra-renal CRP, tubulointerstitial fibrosis, and macrophages in chronic cyclosporine-induced nephropathy in a rat model (Li et al. 2004). Similarly, in rats with spontaneous hypertension that are prone to stroke and develop nephrosclerosis, cerivastatin decreased proteinuria and kidney damage regardless of blood pressure and cholesterol levels (Yamashita et al. 2002). In rats with early renal glomerular infiltrated macrophage, lovastatin inhibited glomerular macrophage infiltration and reduced albuminuria (Park et al. 1998). The suggested nephroprotective mechanisms associated with statins are summarized in Table 3**.**

**Table 3:** Effect of statins on kidney function

Parameters	Pre-clinical/clinical relevance
Estimated glomerular filtration rate (eGFR)	Statins improved the decline in GFR with a tendency toward a reduction in proteinuria (Fried et al. 2001) Simvastatin reduced 24-h excretion of albumin in urine in diabetic patients suffering from hypertension (Yoshimura et al. 1999) Atorvastatin 10–40 mg/day reduced proteinuria and the rate of kidney disease progression in 56 patients with CKD, proteinuria, and hypercholesterolemia Atorvastatin 10 mg/day or 80 mg/day showed improvement in eGFR with the benefit of atorvastatin 80 mg was considerably greater than that of 10 mg, indicating a dose-related effect
Creatinine Clearance (CrCl)	The usage of atorvastatin, pitavastatin, or pravastatin was associated with higher creatinine clearance, in contrast to control (Zhang et al. 2016) Creatinine clearance rose by 12% in atorvastatin treated patients, compared to 4.9% in the “usual care” patients. Nonetheless, people in both groups who had never used statins or had stopped taking them noticed a 5.2% decrease in creatinine clearance (Athyros et al. 2004)
Albuminuria	Statins do not decrease the progression of renal disease to its last state, although they do lower albuminuria and clinical death (Zhang et al. 2016) Meta-analysis found that statins lower proteinuria and albuminuria (Douglas et al. 2006) Statins lower the albuminuria in patients with diabetic nephropathy (Shen et al. 2016) In a model of immune-mediated glomerulonephritis, pretreatment with simvastatin for six days showed marked improvement in proteinuria (Christensen et al. 2006)
Proteinuria	Atorvastatin medication lowers proteinuria in CKD patients (Bianchi et al. 2003) Pravastatin (10 mg/d) for six months decreased proteinuria in individuals with well-controlled hypertension who have normolipidemic proteinuria by 54% as compared to a placebo (Lee et al. 2002) Simvastatin (20 mg/d) for ten months to microalbumin-uric hypertensive patients with type-2 diabetes reduced proteinuria (35%). The effect seemed to be unrelated to decreases in LDL levels as patients receiving cholestyramine (6 gm/3times/d) did not experience the same benefit (Seliger et al. 2002)

CKD, chronic kidney disease; LDL, low density lipoprotein.

According to studies, lipid-lowering medications may help CKD patients maintain their renal function, and dyslipidemia could be a major factor in the development and advancement of CKD. Furthermore, supplementary or post hoc analyses have looked at other indicators, such as renal function, even though the main goal of the historic statin trials has been to reduce CV risk (Mitrofanova et al. 2023). Instead of concentrating on the mesangial cell, a reported mechanism of statin-induced kidney protection targets the podocyte. Damaged podocytes result in glomerulosclerosis and proteinuria. In contrast to glomerulosclerosis, the glomerulus can be healed depending on the degree of podocyte injury (podocyte depletion hypothesis). In animal models, statins reduce podocyte apoptosis and mitigate podocyte damage, which may lessen glomerulosclerosis. Cerivastatin medication reduced the quantity of podocytes detected in the urine in a small, randomized study of individuals with chronic glomerulonephritis, while the placebo group showed no change (Haynes et al. 2014; de Zeeuw et al. 2015).

#### End-stage renal disease (ESRD)

In a randomized investigation of hypercholesterolemic patients, simvastatin elevated serum albumin levels and lowered plasma CRP levels as compared to no therapy (Chang et al. 2002). Simvastatin or atorvastatin were administered at random to 28 hemodialysis patients with ESRD who had borderline decreased HDL cholesterol levels along with normal LDL and total cholesterol (TC) values. The results demonstrated that both statins reduced the amounts of residual lipoprotein cholesterol and oxidized LDL, but they had no effect on CRP levels during an 18-week experiment. In a two-year retrospective analysis of data from 3,916 ESRD patients, the US Renal Data System’s Dialysis Morbidity and Mortality Wave 2 study discovered that statin use was linked to a decrease of about one-third in all-cause and CV mortality in dialysis patients. The CVD-specific mortality rate was 70 per 1,000 person-years for statin users and 90 per 1,000 person-years for nonusers (Seliger et al. 2002).

### HMG-CoA reductase inhibitors and central nervous system (CNS) disorders

#### Overview

Studies have been unveiling the striking features of statin drugs family, which far exceed their original intended application, i.e. hypercholesterolemia and coronary heart disease (CHD). Having demonstrated ramified pharmacological activities, statins have shown additional promise in different CNS-related disorders, ranging from strokes to neurodegenerative diseases and brain tumors (Cucchiara and Kasner 2001; Aznaouridis et al. 2019). Another pharmacokinetic property that stands behind the favorable use of statins to target the brain is the lipophilicity of most members, namely, atorvastatin, fluvastatin, lovastatin, pitavastatin, and simvastatin, when compared to the more hydrophilic ones, i.e. pravastatin and rosuvastatin (Climent et al. 2021). While simvastatin and lovastatin are classified as “natural statins”, others, like rosuvastatin, are purely synthetic (Al-Kuraishy et al. 2023a). We will outline the major applications of statin therapy in CNS fields from a pharmacological perspective, highlighting not only potential benefits, but also possible drawbacks and rung alarms.

#### Acute ischemic stroke

Ischemic stroke basically results from partial or complete focal blockade of cerebral blood flow, in which hypertension and hypercholesterolemia play a significant role. The classical explanation behind the beneficial effect of statins in ischemic strokes is the stabilization of cerebral atherosclerotic plaques (Beltrán Romero et al. 2021; Zhao et al. 2023). However, this effect results from the cooperation of different pharmacological mechanisms exerted by statins. The process of atherogenesis is slow and long-term with two major events, lipid deposition and vascular smooth muscle cell migration/proliferation. In vascular macrophages, statins undoubtedly inhibit cholesterol accumulation and MMPs activity, thus hindering the formation of “foam” cells—the early markers of atherosclerosis. It is also believed that statins have anti-platelet activity by means of reducing platelet membrane cholesterol contents, which restricts their aggregation and thromboxane production (Morotti et al. 2022; Zhao et al. 2023).

Oxidative stress is an early and continuous scenario in the process of atherogenesis, for it is associated with plaque formation/destabilization, vascular smooth muscle cell remodeling, and intimal thickening (Zhao et al. 2023). Statins are now confirmed as inhibitors of NADPH oxidases, in particular the first and the second isoforms (Walker et al. 2021; Morotti et al. 2022). NADPH oxidases are major sources of superoxide anion production both in vasculature and in the brain, and they have a leading role in endothelial dysfunction. Of note, statin members that have been proven to inhibit NADPH oxidase enzymes are atorvastatin, fluvastatin, simvastatin, and rosuvastatin (Malfitano et al. 2014). Importantly, the inhibition of NADPH oxidase enzyme has further consequences that exceed restriction of oxidative stress. For example, p38 mitogen-activated protein kinase (MAPK), the inflammation master is activated downstream of NADPH oxidase activity. Accordingly, statins have been shown to protect against p-38 MAPK-mediated inflammation and apoptotic cell death (Jo et al. 2021). On the other hand, some, but not all, statin members have shown a stimulating effect on the antioxidant enzymes heme oxygenase, catalase (CAT), superoxide dismutase (SOD), and glutathione peroxidase (GPx) (Safakheil and Safakheil 2020).

Significant studies have investigated how statins positively influence eNOS enzyme which not only has a role in vascular function, but also in neuroprotection (Zhao et al. 2023). Statins outstandingly increase eNOS expression, activity and enzyme coupling, and thus, NO bioavailability. This effect is believed to arise from statin-mediated inhibition of Rho GTPase signaling in a mevalonate-dependent approach (Malfitano et al. 2014; Zhao et al. 2023). In addition, statins are believed to promote and activate the major metabolic sensor, AMP-activated protein kinase (AMPK), which enhances eNOS phosphorylation at Ser1177. In the same context, upon AMPK activation, cellular autophagy is enhanced, which mitigates ischemic damage to neurons and improves stroke outcome (Carloni and Balduini 2020). Moreover, a statins’ major advantage in ischemic strokes is promoting angiogenesis. However, one should consider that this outcome is only feasible with low doses that are claimed to be clinically relevant. The suggested mechanism behind the pro-angiogenic effects of statins is phosphoinositide 3-kinase (PI3K)/Akt pathway stimulation and subsequent eNOS activation as well as VEGF upregulation (Yang et al. 2021; Zahedipour et al. 2022). A recent study has revealed the role of the TGF-β signal activation by atorvastatin in the process of angiogenesis (Yang et al. 2021).

MMPs, as previously mentioned, are proteolytic enzymes that significantly participate in the pathology of stroke and other neurodegenerative disorders (Yang et al. 2021). Scientific evidence has proven the ability of statins to inhibit MMPs production and activity. Notably, MMP-9 is the most studied isoform that can be restrained by statins. MMP-9 is released due to ischemia by neurons as well as reactive astrocytes and microglia. Upon production, MMP-9 weakens the blood–brain barrier with hazardous outcomes (Malfitano et al. 2014; Bagheri et al. 2020a). Interestingly, simvastatin, by means of inhibiting Rho signaling, could interrupt MMP-9 activation following tissue plasminogen activator therapy that is used to restore blood flow in ischemic strokes. On top of that, simvastatin offered neuroprotection via restriction of both MMP-9 and TNF-α with consequent anti-inflammatory outcomes. Interestingly, in this study, simvastatin was believed to modulate the N-methyl-d-aspartate receptor which is also strongly associated with Parkinson’s disease (PD) (Yan et al. 2011).

On a side note, the recently discovered programmed cell death, ferroptosis, is currently linked to stroke events, and its inhibition could be a promising target for stroke therapies. Nonetheless, statins are so far considered ferroptosis inducers, which might be beneficial in cancer. Surprisingly, statins are thought to activate ferroptotic cell death through inhibition of selenoprotein synthesis via the mevalonate pathway, as well as inhibition of GPx4 activity (Costa et al. 2023). The latter claim about GPx contradicts previous findings about statins and their antioxidant potential. However, studies on ferroptosis are still in the cradle and continuous research would unveil the role of statins in this type of cell death.

Contrariwise to the view of pleiotropy, Salvatore and colleagues disclosed a different interesting conclusion, that total and LDL cholesterol lowering using any lipid-lowering pharmacotherapy cuts down the risk of stroke. Moreover, the authors stated that the stroke risk reduction corresponds also to the extent of cholesterol reduction, and that there is no place for the so called “pleiotropic effects” in such benefit (Salvatore et al. 2020). Another ambiguous finding from animal studies is stated in Christophe’s and colleagues’ meta-analysis. The authors confirmed the superiority of pravastatin over all statins in improving functional outcomes in animal models of ischemic stroke (Hackam and Hegele 2022).

There is a promising agreement among studies that statin therapy, particularly statin pre-treatment, is associated with reduced physical disability following an ischemic stroke event (Ishikawa et al. 2016; Malhotra et al. 2019). Additionally, post-stroke statin continuation is highly encouraged to decrease the risk of stroke recurrence (Lee et al. 2017). However, it seems that all statin members are on par with each other in achieving such benefits in stroke patients; again, raising questions about the superiority of lipophilic members (Tramacere et al. 2019). Hackam and Hegele reported that statins decrease the risk of ischemic stroke and pose no risk on hemorrhagic stroke (Hackam and Hegele 2019). Although there is no solid evidence about the role of statins in primary stroke prevention nor during the acute attack (Beltrán Romero et al. 2021), there are many outstanding clinical trials that confirmed the significant influence of statin therapy against stroke recurrence (2002; Amarenco et al. 2006, 2020a, 2020b). Importantly, all these benefits were mainly attributed to the LDL lowering effect of statin therapy (Beltrán Romero et al. 2021).

#### Parkinson’s disease (PD)

PD ranks second among all neurodegenerative disorders only after Alzheimer’s disease (Al-Kuraishy et al. 2023a). The coincidence of statin intake in PD patients is empirically high. Because PD is plausibly linked with ageing, and due to the brain permeability to most statin members, it is important to assess the effect of statins on PD pathogenesis and progression. Again, the possible impact of statins on PD pathology and phenotype is rather vague. Likewise, the role of cholesterol and other plasma lipids on PD pathology is debatable. Although the neuroprotective credentials of statins in PD are mainly attributed to regulating inflammatory pathways, prolonged statin therapy and the associated lipid lowering effect can backfire on neurons in PD.

Research has been revealing the possible connection between cholesterol and PD pathophysiology. Both brain contents and plasma concentrations of cholesterol have drawn the attention to study their diagnostic and prognostic values in PD (Al-Kuraishy et al. 2024).

Elevated serum lipids have been correlated with propagation of oxidative stress and accumulation of α‐synuclein proteins, the hallmark of PD pathology, in dopaminergic neurons of the substantia nigra (Galper et al. 2022). Meanwhile, various studies have highlighted that statins possess neuroprotective capabilities against PD—an effect that is assumed to be independent of their chief lipid lowering outcomes (Eriksson et al. 2017b; Al-Kuraishy et al. 2023a, 2024). Interestingly, simvastatin, the most lipophilic statin member, has shown supreme efficacy against PD in animal investigations (Roy and Pahan 2011). Besides the well-validated anti-inflammatory and antioxidant mechanisms, statins were found to suppress the degeneration of dopaminergic neurons in the most critical brain region involved in PD; the substantia nigra (Yan et al. 2011; Eriksson et al. 2017b; Dai et al. 2021). This latter effect is thought to arise through activation of the sterol regulatory element binding protein (Eriksson et al. 2017a). Likewise, lovastatin was found to hinder methyl-phenylpyridinium‐induced apoptotic cell death in a dopaminergic neuroblastoma cell line by attenuating oxidative stress. In the same study, however, lovastatin did not affect lysosomal membrane cholesterol content, thus, protecting against lysosomal membrane leakage (Eriksson et al. 2017a). Similar results were achieved with simvastatin in vivo in a rat model of PD (Tan et al. 2016).

Microglial cells are additional culprits in PD scene. These cells are the primary defense line in the CNS and, under normal conditions, are basically dormant. However, various factors can transform microglia into the active phenotype. When activated, microglia strongly participate in PD neuroinflammation by means of releasing different proinflammatory mediators (Isik et al. 2023). Outstanding evidence has verified that statins can suppress microglial activation, inflammatory cytokines production, and, consequently, neuroinflammation (Bagheri et al. 2020b; Bahmad et al. 2021). One suggested mechanism through which statins are thought to inhibit microglial activation is the interruption of NF-κB signaling pathway. These findings stand behind the rationale for perceiving statins as positive adjuncts for PD patients with hypercholesterolemia (Lu et al. 2019).

On the contrary, noxious effects of statins in PD were also experimentally investigated. It has been thought that these harmful consequences are attributed to the direct effect of statins on neuronal homeostasis, rather than plasma lipid lowering (Jeong et al. 2021). In a mouse model of PD, both atorvastatin and simvastatin failed to prevent dopaminergic neuronal death induced by methyl-phenyl-tetrahydropyridine, and both had unfavorable outcomes (Al-Kuraishy et al. 2023a). One suggested mechanism for statin-induced direct neurotoxicity is through the reduction of co-enzyme Q10 which has a pivotal role in stabilizing the mitochondrial membrane and preventing apoptotic as well as ferroptotic cell death. Noteworthy, this is the same mechanism through which statins cause myopathy (Al-Kuraishy et al. 2023a; Bagheri et al. 2023; Bartošová et al. 2023; Costa et al. 2023).

The role of plasma lipids in PD pathology still represents a controversial point, which also raises another query whether the perceived positive effects of statins in PD are cholesterol‐dependent or independent (Yan et al. 2019; Dai et al. 2021; Jeong et al. 2021; Al-Kuraishy et al. 2023a). While Hu and colleagues suggest that hypercholesterolemia may increase the risk of PD in people < 55 years old, various reports have illustrated the good impact of plasma lipids on PD risk (Al-Kuraishy et al. 2023a). Furthermore, although plasma cholesterol levels do not necessarily correlate with brain cholesterol contents, different studies have disclosed a direct relation between plasma lipids, particularly cholesterol, and PD risk (Paul et al. 2015; Dai et al. 2021). Nonetheless, Gudala et al*.*’s meta-analysis yielded a different conclusion; that no link exists between plasma cholesterol levels and the risk of PD (Gudala et al. 2013).

In the light of statin therapy, different studies have unveiled their favorable impact on PD risk and disease progression, and that long‐term statin therapy has the most positive impact on PD (Palermo et al. 2021; Lewis et al. 2022). Additionally, lipophilic statins are plausibly more efficient than the hydrophilic ones in the course of PD, where hydrophilic statins are likely to be neutral (Dai et al. 2021; Lewis et al. 2022). Interestingly, Yan and colleagues reported that atorvastatin is the leading statin member in reducing PD risk (Yan et al. 2019).

However, studies that disagree with the above-mentioned assumptions are to be considered. These opposite studies gathered evidence to attain a conclusion that statin therapy may increase the risk of PD. Given the fact that cholesterol underpins neuroprotection in the brain, thus statins, specially the lipophilic ones, may increase the risk of PD (Jeong et al. 2021). Moreover, the longer the duration of statin therapy, the higher the risk of PD development (Kim et al. 2022). It has been demonstrated that statins decrease the expression and availability of dopamine transporters in the substantia nigra of PD patients (Jeong et al. 2021). Like other monoamine transporters, brain cholesterol content is essential for normal functioning of dopamine transporters (Walker et al. 2021; Al-Kuraishy et al. 2023a). In addition, cholesterol has a central role in maintaining synaptic stability which is also important for cognitive function of PD patients (Fukui et al. 2015; Al-Kuraishy et al. 2023a; De Giorgi et al. 2023).

Nevertheless, some of these studies have drawbacks that can defy the conclusion that statins are directly related to PD risk and/or progression. What further weakens this conclusion is the presence of some findings that illustrate the stronger contribution of hydrophilic than that of the lipophilic ones in PD pathology, which was confirmed through clinical and imaging results. Interestingly, it is believed that genetics contribute to the variabilities in patients’ responses towards the lipid lowering, neuroprotective, and adverse effects of statin therapy.

#### Major depressive disorder (MDD)

The relationship between statins and MDD is probably the most studied among all psychiatric disorders. Despite the significant number of pre-clinical and clinical studies, the outcomes are rather conflicting. While some researchers advocate for the beneficial effects of statins in depressed patients, many others defy this possibility (Walker et al. 2021).

The neuro-immune hypothesis of MDD stands now at the frontlines of depression research (Avan et al. 2021). Accordingly, many drugs with anti-inflammatory potential have been investigated for their anti-depressant properties, including non-steroidal anti-inflammatory drugs (NSAIDs), N-acetylcysteine, different anti-diabetic medications, and statins (Avan et al. 2021; Walker et al. 2021; Muhammad et al. 2024).

One of the valid mechanisms attributed to the anti-depressant effects of statins is RhoA/Rho kinase (ROCK) signal inhibition. ROCKs have been implicated in different neurodegenerative disorders, stroke, and probably psychiatric diseases. In the same context, statins were found to outstandingly inhibit Rho signaling in different disorders (Zhou et al. 2022).

In another study on ovariectomized female rats, simvastatin was found to suppress various inflammatory pathways within the brain, including the P2X7 receptor and the NOD-like receptor-, leucin-rich repeat-, and pyrin domain-containing protein 3 (NLRP3) inflammasome axes. More interestingly, this study reported significant elevation in hippocampal estrogen receptor (ER) expression in rats when treated with simvastatin (Menze et al. 2021). Moreover, Chen et al. (Chen et al. 2023) demonstrated that lovastatin pre-treatment alleviates depressive behavior in rats following traumatic brain injury. The authors attributed this beneficial effect to statin-mediated liver kinase B1 (LKB1)-AMPK pathway activation. Also, in the context of neuroinflammation, simvastatin was found to offer neuroprotection and anti-depressant effects to oppose γ-radiation-induced brain injury in rats. The identified mechanism in the latter study was modulation of indoleamine 2,3-dioxygenase/kynurenine pathway driven by simvastatin treatment. Through the regulation of this pathway, statins can effectively suppress various inflammatory reactions in the brain, hinder neurotoxicity, and enhance serotonin (5-HT) metabolism (Thabet et al. 2021).

Importantly, monoamine deficiency, the most prevailing theory of MDD, can’t be neglected in the context of statins anti-depressant action. Specifically, considering the serotonergic system, mixed results have been reported. Of particular interest, both enhancement of serotonin transporter (SERT) availability (Chen et al. 2024b) as well as reduced SERT activity (De Giorgi et al. 2023) have been found to alleviate depressive symptoms by statins. In fact, it has been suggested that cholesterol is essential for a sound serotonergic system function (Walker et al. 2021). Therefore, extremely low levels of TC probably cause destabilization of SERT and impairment of 5-HT_1A_, 5-HT_3_, and 5-HT_7_ receptors function (Walker et al. 2021; De Giorgi et al. 2023). A similar controversy was seen with simvastatin. Enhanced 5-HT re-uptake and augmented SERT activity via both cholesterol-dependent and independent pathways, which ultimately result in a decrease in 5-HT activity have been described with simvastatin. Meanwhile, animal models have ascribed the anti-depressant properties of simvastatin to the enhancement of hippocampal 5-HT (De Giorgi et al. 2023). In an indirect approach, statins have been reported to regulate the serotonergic effects of standard anti-depressants via the tyrosine kinase receptor (TrkB) of the brain-derived neurotrophic factor (BDNF), the chief moderator of neuroplasticity and resilience (Casarotto et al. 2021).

Although cumulative evidence suggests that statins may have a promising role in the treatment of depression, some concerns have been raised for the possibility of increased suicide and mood deterioration linked to statin treatment (Molero et al. 2020; Ye et al. 2023).

For example, it has been denied that statins are linked to MDD worsening in patients with acute MI (Al Badarin et al. 2013). Another study yielded comparable results, where statins did not have any direct impact on the risk of MDD. The authors also concluded that both users and non-users of statins have the same risk of developing MDD (Köhler-Forsberg et al. 2019). However, one study assumed that only simvastatin can be associated with a moderate, yet statistically significant, increase in the risk of MDD (Dave et al. 2018). It has been observed that traumatic brain injury patients with hyperlipidemia without statin therapy have a higher risk of developing depression than those who have normal plasma lipids. Accordingly, it has been suggested that statin treatment positively impacts traumatic brain injury and its consequences, as well as the pre-existing hyperlipidemia (Chen et al. 2023). In another cohort study on Danish patients (Köhler et al. 2016), combined treatment with statins and selective serotonin re-uptake inhibitors (SSRIs) significantly reduced the frequency of hospital contacts due to depression, when compared to sole SSRI therapy. Noteworthy, co-treatment with statins and SSRIs did not worsen all-cause mortality as compared to SSRIs alone. Similar favorable outcomes on depressive symptoms were achieved with combined citalopram/atorvastatin, fluoxetine/lovastatin, and fluoxetine/simvastatin treatments in MDD patients (Ghanizadeh and Hedayati 2013; Haghighi et al. 2014; Gougol et al. 2015; Avan et al. 2021). Additionally, simvastatin was claimed to improve MDD symptoms more rapidly and more effectively than atorvastatin, which might be attributed to the higher lipophilicity and better brain penetration of simvastatin (Abbasi et al. 2015). Strikingly, anti-inflammatory augmentation therapy, including statins, was recommended by Quinn and co-workers in youth with moderate-to-severe depression (Quinn et al. 2018).

Hence, it can be concluded that the outcomes of statin therapy mostly depend on patients’ characteristics. Therefore, patients with underlying inflammatory disorders, e.g. CVD, are most likely to benefit from statin therapy for their mood and emotions. On the other hand, outcomes of statin use in individuals with no baseline inflammatory pathology are still ambiguous.

#### Schizophrenia

Schizophrenia or psychosis is a mental disorder that entails one’s thoughts, behavior, and emotions. These disturbances are generally referred to as positive and negative symptoms of schizophrenia. The pathophysiology of schizophrenia is complicated, which explains the diversity of the available pharmacotherapies that target different receptors and neurotransmitters within the brain. However, the story has not come to an end and a significant number of patients fail to respond to the standard available therapies.

Statins have been incorporated in schizophrenia research owing to their anti-inflammatory and antioxidant properties specifically, where simvastatin has been the most extensively studied member (Stamu-O'Brien and Kroumpouzos 2023; Peng et al. 2024). Accumulating evidence has been revealing the role of different inflammatory cytokines, particularly TNF-α and IL-1β, in the pathogenesis of schizophrenia and worsening of symptoms (Peng et al. 2024). Besides, schizophrenia patients probably have higher microglial and T-cell activity when compared to general population (Postolache et al. 2021; Sato et al. 2022).

Despite the mixed results of clinical trials (Avan et al. 2021), statins, especially simvastatin, seem to stand at the positive side for schizophrenia patients. It makes sense that this class of medications can be valuable as added on therapy in schizophrenia, given that antipsychotics, particularly the second-generation ones, are associated with many metabolic and cardiovascular adverse effects (Pillinger et al. 2020; Avan et al. 2021). Meanwhile, some researchers believe that hyperlipidemia arises as part of the pathophysiology of schizophrenia (Chen et al. 2024a). Moreover, it has been found that schizophrenia patients can develop early-onset CHD when compared to normal population (Lambert et al. 2022).

Of note, many animal and in vitro studies have also emphasized the superior role of simvastatin over other statin members, however, most of them did not focus on schizophrenia but rather evaluated a general neuroprotective role for simvastatin (Peng et al. 2024). Accordingly, one can’t detach statins from their main lipid-modifying function when addressing schizophrenia. In fact, emerging studies have focused on abnormalities of lipid metabolism as a pathological mechanism in schizophrenia. Research has identified derangements in sphingomyelin, phospholipids, free fatty acids, and phospholipase A2 enzyme activity in schizophrenia patients (Stamu-O'Brien and Kroumpouzos 2023).

In what is called “The membrane hypothesis of schizophrenia”, abnormal phospholipid metabolism in both neurons and red blood cells is postulated (Li et al. 2022; Stamu-O'Brien and Kroumpouzos 2023). In this context, lower levels of plasma free fatty acids in newly diagnosed schizophrenia patients were reported, as compared to the controls. The authors owed this abnormality to the increased phospholipase A2 enzyme activity, which leads to more breakdown of plasma membrane phospholipids. Remarkably, in these patients, antipsychotic treatment led to normalization of their plasma free fatty acids levels. Because free fatty acids have pivotal roles in neurotransmission, prostaglandins production, and many other vital processes, statins and omega-3 fatty acids are currently investigated in the sense of the “membrane hypothesis”. Another explanation to the membrane hypothesis was built based on schizophrenia-associated oxidative stress, where oxidants cause plasma membrane phospholipid remodeling and subsequent membrane degradation (Stamu-O'Brien and Kroumpouzos 2023). In the current mini review, we will focus on the findings of clinical trials due to the limitation of the pre-clinical ones.

Lipophilic statins were reported to improve negative symptoms of schizophrenia (Avan et al. 2021), with no substantial effect on either positive symptoms or the so called “general psychopathology symptoms”. Simvastatin impact, however, exceeded the negative symptoms and improved overall symptoms of schizophrenia (Peng et al. 2024). Interestingly, a single study reported that hydrophilic statins can improve schizophrenia positive symptoms. In addition, it was recognized that schizophrenia statin users (either hydrophilic or lipophilic) had lower risk of psychiatric hospitalization when compared to non-user counterparts (Postolache et al. 2021). Similarly, a nationwide Taiwanese study revealed that not only statins, but also fibrates reduce the risk of mortality in schizophrenia patients (Chen et al. 2024a). Another study on Taiwanese schizophrenic patients showed that occurrence of dyslipidemia in the early course of the disease is associated with increased risk of overall mortality (Hsu and Ouyang 2021). On the other side, Chen and colleagues (Chen et al. 2024c) denied any genetic association of cholesterol metabolism abnormality with the risk of schizophrenia. Likewise, another study found no correlation between plasma cholesterol levels and aggression/ impulsivity in schizophrenia patients (Hjell et al. 2020). Finally, in a very unusual outcome, some authors showed that the use of statins does not decrease the risk of cardiovascular disease in schizophrenia patients after 12 months but potentially decreases psychiatric admissions (Avan et al. 2021).

In the light of neuroinflammation and neuro-immune theory of schizophrenia, various pre-clinical and clinical studies have evaluated the utility of different anti-inflammatory agents and immune modulators in schizophrenia (Fond et al. 2020). However, it has been implied that anti-inflammatory treatment is conditionally beneficial in case of exaggerated baseline inflammation. Also, Fitton et al*.* (2022) has reached a conclusion that anti-inflammatory agents that seem beneficial in affective disorders (celecoxib, pioglitazone, & statins) may differ from those that can be effective in schizophrenia (minocycline & aspirin), which suggests a more complicated mechanism of action for these drugs than merely fighting inflammation. In a recent interesting study (Zaki et al. 2024), the authors aimed to spot biomarkers that can predict treatment response in new-onset schizophrenia patients exposed to simvastatin. The authors linked changes in insulin receptor signaling in B-cells treated ex vivo with simvastatin to cognitive improvement upon drug administration. Besides the authors’ main aim in identifying feasible biomarkers, they pinpointed to the role of B-cells and insulin signaling in the pathogenesis of schizophrenia and how simvastatin can modify their abnormalities. In addition, hyperactivity and proliferation of T-cells, accompanied by production of proinflammatory cytokines, have been recognized in the CNS of schizophrenia patients (Schlaaff et al. 2020; Sato et al. 2022). Remarkably, it was revealed that T-cell hyperactivity is associated with overexpression of the delayed rectifier potassium channels. In fact, the activity of these potassium channels can be suppressed by a wide range of drug classes, including statins, and hence, hindering the inflammatory response (Sato et al. 2022).

Contrariwise, negative reports with anti-inflammatory therapy in the setting of schizophrenia can’t be overlooked. In a large 12-week randomized controlled trial, both naproxen and simvastatin, as well as their combination failed to demonstrate any positive impact on schizophrenia symptoms (Weiser et al. 2023). Sommer and colleagues also achieved similar results with simvastatin (Sommer et al. 2021). These cumulative outcomes make the clinical community skeptical about the use of statins to improve psychiatric symptoms of schizophrenia.

To replenish our hopes, some researchers suggest that statin therapy is more beneficial in stabilized schizophrenia patients, and that higher doses and sufficient duration are required in clinical trials than those applied in animal studies (Avan et al. 2021).

### HMG-CoA reductase inhibitors and respiratory complications

#### Overview

Recent studies show other important outcomes, such as their anti-inflammatory effects, antioxidant properties, and immunomodulatory effects, serving other indications in pulmonary diseases. These multifaceted approaches open up opportunities for investigation into the treatment of some difficult respiratory diseases through inflammation, oxidative stress, and immune dysregulation (Saadat and Boskabady 2021). Currently, the findings focus on the mechanisms of action, therapeutic relevance, and issues related to the use of these drugs in respiratory health and provides a thorough outline of present findings, limitations and challenges of statins use.

#### Statins and respiratory function: molecular perspectives

Statins inhibit the mevalonate pathway as they inhibit farnesyl and geranylgeranylation of Rho and Ras, which regulate inflammatory signaling pathways, thereby reducing the production of isoprenoid intermediates (Wang et al. 2024). These intermediates are essential for the post-translational modification of signaling proteins such as Rho and Ras, with respect to their regulatory effects on the inflammatory pathways; therefore, by modulating these pathways, statins will inhibit the production of proinflammatory cytokines including IL-6, TNF-α, and IL-1β, which are crucial intermediates in respiratory inflammation (Sheridan et al. 2022b; Sadeghi et al. 2024). By reducing inflammation, this causes relief in symptoms of local respiration as well as preventing systemic inflammatory responses related to severe respiratory diseases. The action of statins is therefore capable of targeting these pathways; thus, they become potential adjunctive therapy in inflammatory lung disorders (Zhang et al. 2022).

Statins promote the expression of antioxidant enzymes including that of SOD and diminish the production of ROS in endothelial cells (ECs). Their combined action helps to address oxidative damage to the lungs, which is commonly seen in a range of respiratory diseases (Salama et al. 2024). The reduction of oxidative stress is of particular importance in diseases such as chronic obstructive pulmonary disease (COPD) and asthma because oxidative injury contributes to the progression of the disease and worsens clinical outcomes (Vasconcelos et al. 2021). Statins can thus help maintain cellular redox balance, thus contributing to the preservation of tissues and restoration of function in respiratory conditions with oxidative stress (Bradbury et al. 2018).

Statins improve the condition of the endothelium and enhance the bioavailability of NO; both of these mechanisms play a role in reducing vascular inflammation and pulmonary hypertension, which typically occur in the respiratory complications (Rysz-Górzynska et al. 2016). Improved integrity in endothelial tissues reduces risk of vascular leakage, improvements in blood flow, and even increase the availability of oxygen to the tissues, which is crucial in the management of pulmonary hypertension and acute respiratory conditions (Han et al. 2020). Statins are assumed to perform endothelium-stabilizing roles clearly show their importance against secondary complications such as hypoxia and thromboembolic events in severe respiratory diseases (Chen et al. 2017).

Statins showed immunomodulatory effects, where adaptive immune responses are modulated with the suppression of T-cell activation and maintenance of regulatory T-cell development (Shahbaz et al. 2019). These are acts of maintaining the immune balance and preventing excessive immune responses that contribute to tissue damage (Alabed et al. 2022). This role is important in health conditions such as asthma and pulmonary fibrosis, wherein immune dysregulation plays an essential role (Thomson 2017; Kim et al. 2021c). Additionally, statins can modulate the action of innate immunity, thereby enhance infection control and decreasing related unfavorable outcomes. The immunomodulatory effects of statins place these drugs on the list of promising treatments for immune-mediated respiratory diseases and systemic inflammatory syndromes (Alsanosi and Alshanberi 2023). The suggested mechanisms of statins influence in respiratory disorders are displayed in Table 4**.**

**Table 4:** Defensive mechanisms of statins in respiratory disorders

Disorder	Molecular mechanisms
COPD	Statins reduce systematic inflammation markers such as CRP in COPD patients, leading to lesser frequency and severity in the exacerbation of diseases Statins modulate immune responses and reduce oxidative stress in the respiratory tract
Asthma	Statins suppress eosinophil activity and their cytokinesis, which are primary elements of airway inflammation and hyper-responsiveness in asthma, resulting in reduction in symptoms such as wheezing and shortness of breath Statins show potential beneficial effects in combination with corticosteroids in patients with refractory corticosteroid-resistant asthma (Kim et al. 2021b)
Pulmonary arterial hypertension	Statins reduce vascular remodeling and smooth muscle cell division in pulmonary arteries, enhancing hemodynamic parameters such as right heart catheterization and pulmonary vascular resistance, a pivotal contributor to pulmonary hypertension progression (He et al. 2023)
Idiopathic pulmonary fibrosis	Statins slow the fibrotic progression through reducing the activity of pro-fibrotic cytokines TGF-β in the lung (Dolivo et al. 2023) Statins could improve the survival of patients with idiopathic pulmonary hypertension (Kreuter et al. 2017)

COPD, chronic obstructive pulmonary disease; CRP, C-reactive protein; TGF, transforming growth factor.

#### Infectious respiratory diseases

Interestingly, latest reviews during the covid-19 pandemic clarify the potential of statins for alleviating the hyper-inflammatory responses during severe cases of the disease (de Mesquita et al. 2024). Mechanistic studies exhibit that statins are cytokine modulators and enhance endothelial integrity reduced the severity of acute respiratory distress syndrome (Mao et al. 2023). Statins might as well further improvement in outcomes, usually seen in thrombotic complications in covid-19 cases. This multifactorial approach underscores that statins have the potential as supplemental therapy for severe viral respiratory infections (Saad et al. 2022).

In addition, it has been determined that statins decrease the risk of pneumonia-related complications because they suppress systemic inflammation and promote immune responses. It can also reduce the progression of respiratory failure that can be seen mostly in vulnerable populations. There could be potential advantages for people who suffer recurrent pneumonia and severe pneumonia through their immunomodulation properties (Zeenny et al. 2020; Yun et al. 2021).

### HMG-CoA reductase inhibitors and autoimmune diseases

#### Overview

Interestingly enough, statins have gained attention as potential agents for manipulating autoimmune diseases. In fact, statins have yielded promising outcomes in immunogenic conditions (Dehnavi et al. 2020). It has been demonstrated that statins can outstandingly inhibit EC activation induced by anti-phospholipid antibodies, suppress autoreactive B-cell activation, mitigate Th1-driven autoimmune responses, and lower serum homocysteine. Likewise, cumulative evidence has highlighted their capacity to cut down high-sensitivity CRP, thus, underscoring their ability to modulate inflammation and maladaptive immune activity (Sahebkar et al. 2016). Of note, convincing findings suggest that statins can impact immunological responses via both mevalonate pathway-dependent and -independent mechanisms (Dehnavi et al. 2020).

#### Statins and rheumatoid arthritis (RA)

RA is a life-long autoimmune disorder that is marked by joint damage and is often accompanied with systemic comorbidities. The basic pathophysiology behind RA is an imbalance in CD4+ lymphocytes, which triggers the release of inflammatory cytokines, thus, contributing to joint tissue damage (de Oliveira et al. 2020). In addition, proinflammatory factors, such as IL-6, stimulate the production of osteoclasts that contribute to joint damage, swelling, and degeneration (Ren and Li 2023).

Statins are emerging as novel modulatory agents for RA pathology due to their immunomodulatory, anti-inflammatory, and antioxidant properties. Studies have shown that different statin members can exert disease-modifying effects in RA, including reductions in inflammatory factors and enhancements in disease severity ratings (van Boheemen et al. 2021). Research has also demonstrated that statins can significantly improve acute-phase reactants, such as CRP and erythrocyte sedimentation rate (ESR), as well as levels of interferon-gamma (IFN-γ), TNF-α, MMP-3, cartilage oligomeric matrix protein (COMP), reduced glutathione (GSH), lymphocytes, ILs, and immunoglobulins (IGs) (Perucha et al. 2019; de Oliveira et al. 2020).

Studies that implemented simvastatin have shown suppression in the expression of rheumatoid factor (RF), MMP-3, and COMP. Additionally, simvastatin has been found to significantly improve serum levels of IgG and antinuclear antibodies (ANA), and to ameliorate oxidative stress (Ahmed et al. 2015). Simvastatin has been shown to reduce TNF-α and other serum inflammatory markers in RA patients. Moreover, in experimental models of collagen-induced arthritis, simvastatin successfully inhibited disease progression and alleviated clinical manifestations, thus, supporting its potential in managing T helper cell 1 (Th1)-driven maladaptive immune responses (Aminifar et al. 2023). As well, research has demonstrated that simvastatin effectively decreases cytokine production and inhibits NF-κB activation in synoviocytes derived from RA patients following stimulation with IL-1β. In the same report, simvastatin improved clinical disease scores by means of increasing the expression of TGF-β while reducing the expression of IL-6, IL-12p40, IL-12p70, RANTES (CCL5), and macrophage inflammatory protein-1β (MIP-1β). These findings were also consistent with lower levels of inflammatory mononuclear cells and weaker Th1/17 cell infiltration. Consistent with the later outcomes, simvastatin could suppress T-cell proliferation in co-cultures with primary microglial cells (Mostafa et al. 2020).

In the same vein, atorvastatin has demonstrated the ability to inhibit the synthesis of various cytokines in peripheral blood mononuclear cell cultures obtained from advanced RA patients. Atorvastatin may also alter immune response and diminish inflammation, thus, potentially mitigating RA severity and its related symptoms (de Oliveira et al. 2020).

#### Statins and multiple sclerosis (MS)

MS is a long-term inflammatory condition of the CNS and is characterized by axonal demyelination and damage. Importantly, peripheral immune cells migrate to the CNS through a compromised blood–brain barrier in the subarachnoid space (Faissner et al. 2019). Acute MS lesions typically contain large numbers of macrophages and CD8+ T cells, with smaller contributions from CD4+ T cells, B cells, and plasma cells. In early stages of relapsing–remitting MS (RRMS), demyelination is mainly confined to focal lesions, while other areas of white matter remain unaffected. Over time, B and T cells infiltrations become more diffuse, resulting in widespread axonal damage and progressive atrophy of both white and gray matter. Progressively, the inflammatory activity becomes sustained by CNS-resident microglial cells (Zéphir 2018).

In experimental autoimmune encephalomyelitis (EAE), statin therapy markedly reduced the formation of CNS lesions and delayed disease onset through exhibiting anti-inflammatory and immune-modulatory effects (Al-Kuraishy et al. 2023b). The beneficial effects of statins in EAE were also attributed to tolerogenic modifications of antigen-presenting cells (APCs), alterations in the Th1 cytokine profile, and suppression of IL-6/23 transcription. Moreover, statins are believed to promote an anti-inflammatory T-cell response, improve cerebral blood flow by modulating NO production, and prevent glutamate-induced excitotoxicity (Abdalla et al. 2021).

Although early research suggested that statins induce a Th2 response, subsequent studies have confirmed that atorvastatin can inhibit antigen-specific T-cell proliferation and can reduce the secretion of IFN-γ, TNF, IL-17, and IL-12. Interestingly, while Th2 induction requires the signal transducer and activator of transcription 6 (STAT6), atorvastatin prohibited EAE even in STAT6-deficient mice that are unable to produce IL-4 by Th2. Furthermore, the effects of statins have been shown to be independent of forkhead box protein P3 (Foxp3+) regulatory T cells (Weber et al. 2014).

Statins have been shown to suppress IFN-γ-induced major histocompatibility complex-II (MHC-II) molecules in unspecialized APCs. For instance, atorvastatin has been able to decrease IFN-γ-induced MHC-II transactivator (CIITA) transcription controlled by the CIITA promoter and to suppress IFN-γ-activated MHC-II molecules in microglia (Sheridan et al. 2022a). Intriguingly, this later outcome could be mitigated by L-mevalonic acid. Of note, the activation of CD4 + T cells necessitates a co-stimulatory signal that is initiated by T cells expressing the CD40 ligand (CD40L) on their surface. The CD40L interacts with costimulatory molecules on APCs, thus, initiating the expression of the co-stimulatory molecules, B7-1 (CD80) and B7-2 (CD86), which subsequently bind to and activate CD28 on T cells. Remarkably, statins can also suppress the expression of many co-stimulatory molecules that are activated by IFN-γ on APCs (Xu and Wang 2022).

#### Statins and systemic lupus erythematosus (SLE)

SLE is a chronic autoimmune disorder signified by the production of auto antibodies and the formation of immune complexes that can affect multiple organs. Notably, failure of self-tolerance is a key factor in the onset and progression of SLE (Tsokos et al. 2016). Dysregulated innate and adaptive immune responses that target self-antigens lead to auto antibody production and immune complex deposition in tissues. This in turn activates the complement system, promotes the recruitment of neutrophils and monocytes, and sustains self-reactive lymphocyte activity, thus, contributing to inflammation and tissue damage (Pan et al. 2020).

SLE presents with a range of clinical manifestations, including an elevated risk of CVD and a higher prevalence of both atherosclerotic and unstable plaques (Frostegård 2023). Investigating the crosstalk between SLE and atherosclerosis can provide valuable insights into the role of immune dysregulation in the development and progression of atherosclerotic disease (Sun et al. 2021).

The pleiotropic effects of statins are largely attributed to the inhibition of isoprenoid synthesis, which impacts multiple signaling pathways (Oesterle et al. 2017). *In-vivo* studies have demonstrated that statins influence nitric oxide production, reduce proinflammatory cytokine levels, suppress vasoconstrictive factors, and modulate platelet reactivity. These actions collectively contribute to reduced inflammation and a lower risk of thrombosis (Pirro et al. 2019). In SLE, statins have been shown to mitigate thrombosis risk and may exert protective effects on atherosclerosis similar to those observed in CAD, including slowing the progression of atherosclerotic plaques and lowering CV risk (Watanabe et al. 2018). Given that CHD and atherosclerosis are currently the leading causes of mortality in SLE, the potential of statins to address these complications highlights their therapeutic value in this population (Mazurek et al. 2020).

Again, statins exhibit significant immunomodulatory and anti-inflammatory effects in SLE that extend beyond their cholesterol-lowering properties. Through inhibiting the ROCK pathway, statins reduce IL-17 and IL-21 levels, suppress proinflammatory mediators, such as IFN-α, TNF-α, IL-6, and IL-8, and, finally, enhance anti-inflammatory cytokines like IL-4 and IL-10 (Ruiz-Limon et al. 2015; Rozo et al. 2017). Importantly, statins can also restore immune cell functionality by different means. These include reversing lipid raft abnormalities in autoreactive T cells, decreasing the C-X-C motif chemokine ligand 9 (CXCL9) expression to limit lymphocyte recruitment towards atherosclerotic plaques, and disrupting Toll-like receptor (TLR) signaling (Houssen et al. 2016). Additionally, statins alter oxidative stress-related gene expression and reduce oxidative stress markers (Moon et al. 2014).

In the light of cardiovascular complications of SLE, statins have been found to decrease atherosclerotic plaque formation and to improve endothelial function via NO enhancement and ROS restriction. In SLE models, statins could also downregulate MHC-II expression on monocytes and B cells, thus attenuating T-cell proliferation. Statins are well-known to inhibit geranylgeranyl transferase and Rho kinase, which diminishes type 1 IFN production through cholesterol-independent mechanisms (Rozo et al. 2017; Khan 2021). These multifaceted actions contribute to reductions in SLE disease activity, which is evidenced by improvements in The Systemic Lupus Erythematosus Disease Activity Index (SLEDAI) scores, decreased organ damage, and improved inflammatory profiles (Tan et al. 2019).

#### Psoriasis

Psoriasis is a chronic relapsing inflammatory skin disorder. However, it is also an immune-mediated systemic disease driven by a complex interplay of genetic and environmental factors (Griffiths et al. 2021; Raharja et al. 2021). Psoriasis is clinically characterized by erythematous plaques covered with silvery-white scales, often accompanied with punctate hemorrhages. The pathogenesis behind psoriasis involves activation of naïve T cells and keratinocytes (KCs) by APCs, which ultimately leads to hyperproliferation of KCs and sustained inflammation (Yamanaka et al. 2021). Psoriasis is incorrectly perceived as skin condition. Nonetheless, it is a systemic disease that frequently coexists with different metabolic disorders, such as obesity and atherosclerosis (Yamazaki 2021; Gonzalez‐Cantero et al., 2023). While its pathogenesis has been extensively investigated in the context of inflammation and immune dysregulation, the exact mechanisms remain incompletely clear. Patients with psoriasis exhibit elevated levels of IL-23 and Th17-associated cytokines, such as IL-17, IL-6, and IL-22, when compared to healthy individuals (Lee and Moon 2023).

Interestingly, psoriasis has been closely linked with lipid metabolism, where psoriasis patients have been found to have abnormal plasma lipid profiles in recent investigations (Hao et al. 2021). Specifically, patients with psoriasis exhibit higher levels of LDL, TG, and TC, along with lower levels of HDL and VLDL, when compared to healthy individuals (Zhou et al. 2024). Recent studies suggest that statins not only regulate plasma lipoproteins and reduce CV risk in psoriasis patients but also exhibit immunomodulatory activities that play a role in improving psoriatic skin lesions. A study by Matwiejuk et al. (Matwiejuk et al. 2023) stated that psoriatic patients are characterized by significant lipid imbalances that are strongly associated with metabolic syndrome and an increased risk of mortality from CVD. Interestingly, the study highlighted a bidirectional association between psoriasis and lipid metabolism, where treating psoriasis can positively affect lipid profiles, while managing lipid disorders can also improve the clinical course of psoriasis.

Studies utilizing 18F-fluorodeoxyglucose positron emission tomography/computed tomography (18F-FDG-PET/CT) have demonstrated that patients with psoriasis exhibit increased aortic vascular inflammation. In addition, the severity of the disease has been linked to aortic inflammation, which is independent of traditional CV risk factors, such as hypercholesterolemia. Notably, Kaiser et al. (Kaiser et al. 2021) have reported that statin therapy is associated with reduction in vascular inflammation among patients with psoriasis. A double-blind, randomized controlled trial (Al Salman et al. 2021) investigated the potential benefits of combining oral simvastatin with Narrowband ultraviolet B (NB-UVB) phototherapy in a group of psoriasis patients. Compared to baseline, patients who were treated with phototherapy ± simvastatin demonstrated a significant reduction in the Psoriasis Area and Severity Index (PASI) scores at 6 and 12 weeks. In 2020, a group of scientists conducted a meta-analysis of randomized controlled trials that investigated the effect of statins on psoriasis severity to assess their therapeutic potential. The results from multiple randomized controlled trials showed that statin treatment significantly reduced psoriasis severity, with notable improvements in both skin lesions and inflammatory markers (Socha et al. 2019). Another recent meta-analysis conducted by a group of scientists in 2024 demonstrated a statistically significant reduction in PASI scores at week 8 following statin treatment (Socha et al. 2019). In 2025, a nationwide cohort study conducted on Korean adults investigated the hypothesis that prior use of lipophilic and hydrophilic statins could influence the onset of psoriasis. The study revealed that statin use is associated with significantly lower risk of developing psoriasis (Han et al. 2024).

In conclusion, statins have been shown to improve both disease severity and quality of life in patients with psoriasis, suggesting the potential therapeutic benefits of statin therapy. By reducing the severity of psoriasis lesions and modulating inflammatory pathways, statins may offer a valuable adjunct treatment for managing psoriasis and enhancing patient outcomes.

### HMG-CoA reductase inhibitors and gastrointestinal disorders

#### Peptic ulcer disease (PUD)

One of the most prevalent gastrointestinal conditions linked to high healthcare costs is peptic ulcer disease (PUD) (Abdullah and Almukhtar 2023). According to (SUNG et al. 2009), a prior study estimated the yearly global incidence rates of PUD to be between 0.1 and 0.2 percent based on physician diagnosis and 0.03 to 0.17 percent based on hospitalization data. NSAID use, Helicobacter pylori infection, alcohol misuse, smoking, and physical stress are among the risk factors for PUD that have been found (Abdullah and Almukhtar 2023). Recent epidemiological research indicates that statin use may reduce the incidence of peptic ulcer disease.

The protective effect of statins against the development of PUD may result from several different mechanisms. The pathophysiology of peptic ulcers is significantly influenced by proinflammatory mediators and ROS. Therefore, taking statins, which are well-known antioxidant and anti-inflammatory drugs, may lessen the inflammation in the stomach mucosa and stop ulcers from forming. Simvastatin pretreatment could lower gastric acidity and the development of gastric ulcers caused by alcohol and indomethacin by altering gastric NO levels, lowering glutathione S-transferase (GST) levels, and raising SOD and CAT levels. This study in rats showed the anti-inflammatory effect of statins on gastric mucosa (Tariq et al. 2007). Another rat study revealed that the level of prostaglandin E2 in the gastric mucosa of rats pre-treated with simvastatin was significantly higher than in rats not exposed to statins (Heeba et al. 2009).

However, there is disagreement over whether statins reduce the risk of PUD in humans due to inconsistent findings from epidemiological studies, which range from significantly lower risk to negligibly higher risk (Badillo et al. 2015; Feng et al. 2015). As a result, more research is still needed to fully describe the risk. In conclusion, several investigations discovered that statin users had a numerically decreased risk of PUD. The findings, however, fell short of statistical significance. To fully characterize this possible protective impact, more research is still needed.

#### Gastroesophageal reflux disease (GERD)

GERD, predominantly a dysfunction of the lower esophageal sphincter, is a prevalent digestive disorder globally, with an estimated prevalence of 18.1–27.8% in North America. The quintessential and prevalent symptom of GERD is heartburn. Heartburn is a burning sensation in the chest that radiates into the mouth, caused by acid reflux into the esophagus (Maret-Ouda et al. 2020).

Statins are medications that were first created to lower blood cholesterol. They are used extensively to lower CV morbidity and mortality since they are safe and effective (Khatiwada and Hong 2024b). Statins have been reexamined and thought to have pleiotropic effects in recent decades. Indeed, statins have been shown to have a net anti-inflammatory effect by influencing inflammatory markers and pathways, including the expression of adhesion molecules and chemokines that attract inflammatory cells, according to experimental investigations conducted in cell cultures and animal models (Ishizuka et al. 2024). Patients with GERD benefited from statins defense mechanisms. Statin use has been linked to milder forms of esophagitis; this suggests that statin use is protective mostly during the early stages of inflammation (Khoury et al. 2019). Thus, this could imply that statins protect against the development of esophagitis in GERD patients. According to a different study, statins can protect against erosive esophagitis by reducing the degree of inflammation and enhancing quality of life (Fujii et al. 2009).

In addition, the preventive capabilities of simvastatin were demonstrated by a mouse model of mixed gastroduodenal reflux disease. The results of this investigation showed that oral administration of simvastatin in vivo led to a reduction in the histologic alterations of the distal esophagus that were caused by reflux. Based on these findings, simvastatin has been identified as a possible prophylactic medication that can limit the development and progression of esophageal damage caused by reflux (Gergen et al. 2022).

#### Ulcerative colitis (UC)

UC is an inflammatory bowel disease (IBD) marked by nonspecific, recurrent inflammation of the gastrointestinal system, with primary symptoms including diarrhea, stomach pain, and weight loss (Gros and Kaplan 2023). The precise etiology of UC is predominantly unclear; nevertheless, various factors, including genetic modifications, disruptions in intestinal microbiota, immunological stress, and injury to colonic mucosa, have been recognized as contributing elements to its pathogenesis (Nakase et al. 2022). Recent studies have shown a significant correlation between dysbiosis of intestinal microbiota and UC (Franzosa et al. 2019; Zheng et al. 2022). Metabolites derived from gut microbiota, including bile acids, short-chain fatty acids, and tryptophan metabolites, significantly contribute to immunological equilibrium, energy metabolism, and the preservation of mucosal integrity (Federici 2019).

Importantly, as previously mentioned, suppression of isoprenoid intermediates generated throughout the mevalonate pathway is responsible for many beneficial outcomes exerted by statins (Oesterle et al. 2017). The structure and functionality of certain cell-signaling proteins, such as the superfamily of small GTPases (Ras, Rho, and Rac) involved in cell trafficking, cell cycle, and cell differentiation, are greatly influenced by these lipid mediators. Therefore, in addition to their role in treating hypercholesterolemia, statins may also be used to treat autoimmune and inflammatory illnesses (Qin et al. 2024).

In an experimental animal model of colitis caused by dextran sulfate sodium, rosuvastatin was found to reduce the degree of intestinal inflammation (Saber et al. 2021). The TLR4/NF-κB signaling pathway may be inhibited by atorvastatin’s anti-inflammatory action on 2,4,6-trinitrobenzene sulfonic acid (TNBS)-induced rat colitis (Rashidian et al. 2016). Additionally, Basso et al. found that PPAR-α, a possible predictive biomarker of therapeutic responsiveness in IBD, is necessary for the positive effects of atorvastatin in colitis (Basso et al. 2021). In 2024, a recent study demonstrated atorvastatin preventive efficacy in a mouse model of UC. According to the study findings, atorvastatin protects intestinal integrity in colitis, most likely via altering the immune response that is mediated by cells rather than by innate immunity. Furthermore, as indicated by body weight loss, diarrhea, and rectal bleeding, atorvastatin dramatically decreased the severity of murine colitis, which in turn led to a lower disease activity index. Additionally, atorvastatin treatment dramatically decreased IL-17 levels, as well as IL-6 (a crucial cytokine). In a domino effect, the proinflammatory cytokine, IL-17, causes fibroblasts, ECs, macrophages, and epithelial cells to produce more proinflammatory molecules (Mageed et al. 2024).

### HMG-CoA reductase inhibitors and bone disorders

Cumulative evidence suggests a bidirectional relationship between lipid/bone metabolism, where some shared mediators influence both atherosclerotic plaque calcification and progression of bone disorders, such as osteo-porosis and -arthritis (Tian and Yu 2015). Disruptions in lipid homeostasis can, on the one side, impair osteoblast activity, and, on the other side, promote osteoclast-mediated bone resorption (Alekos et al. 2020). It has been noted that targeting lipid metabolism with statins can potentially enhance bone quality in metabolic disorders pertinent to the skeletal system, which further underscores the mutual bonds of these metabolic pathways (Kim et al. 2021a).

Besides their cholesterol-lowering outcomes, statins exhibit numerous pleiotropic actions that are LDL-independent (Afshari et al. 2021). In this context, in vitro experiments have demonstrated that statins exert anabolic effects on a range of bone cell lines, including human and murine osteoblast-like cells, as well as human bone marrow stromal cells (hBMSCs) (Zhang et al. 2014). The latter findings suggest that statins, while being primarily used for lipid-lowering purposes, may also positively affect bone metabolism. In the same vein, statins have been found to regulate bone cell differentiation by directly influencing key mediators of osteogenesis. These mediators include alkaline phosphatase (ALP), bone morphogenetic protein-2 (BMP-2), glucocorticoids, MMP-1, TGF-β, and type I collagen. Additionally, statins are believed to exert their effects on bone cell differentiation through multiple molecular pathways, including protein isoprenylation, Wnt/β-catenin, as well as osteoprotegerin/receptor activator of NF-κB and its ligand (OPG/RANKL/RANK) axes. These later signaling cues ultimately modulate the activity of different transcription factors, such as NF-κB, c-Jun N-terminal kinase (JNK), and ERα. Moreover, various in vitro studies have been established to uncover the mechanisms through which statins influence bone cell differentiation (Chamani et al. 2021).

Simvastatin has been extensively studied for its impact on bone metabolic activity. Simvastatin governs different signaling pathways that mediate its osteogenic, anti-osteoblastic, and anti-adipogenic attributes. By means of antagonizing TNFα-driven Ras/Rho/MAPK and PI3K pathways activation, while amplifying BMP-Smad signaling, simvastatin is believed to enhance osteoblast differentiation (Venkatesan et al. 2019). Additionally, simvastatin activates Wnt/β-catenin pathway to promote osteogenesis. Interestingly, in osteoblasts, simvastatin fosters VEGF expression—a critical factor in directing BMSCs towards osteoblast differentiation rather than adipocyte formation (Balmayor 2015). Moreover, simvastatin safeguards osteoblasts against apoptosis via TGF/Smad3 signal interruption, which ultimately suppresses ALP activity. Besides, simvastatin inhibits the mevalonate pathway, which reflects on prenylation of GTP-binding proteins that are essential for osteoblast survival (Moshiri et al. 2016). Furthermore, simvastatin disrupts osteoclastic activity by enhancing the secretion of OPG which competes with RANKL, thereby inhibiting osteoclast maturation. These multifaceted actions give credentials to simvastatin as a potential therapeutic agent for repairing bone defects and managing osteoporosis (Soares et al. 2018). The promising effects statins in bone disorders are summarized and displayed in Table 5**.**

**Table 5:** Role of statins in bone diseases

Members	Molecular mechanisms
Simvastatin	Reduced the expression of cartilage-degrading enzymes and the proinflammatory cytokine IL-1β, and enhanced the expression of type II collagen together with the autophagic marker, microtubule-associated protein 1 light chain 3 (LC3), in articular cartilage during early stages of inflammation (Tanaka et al. 2019) Alleviated osteoarthritic changes and modulated joint structures to counteract monoiodoacetate (MIA)-induced damage in a rat model of temporomandibular joint (Abdelhameed et al. 2020) Suppressed MMP expression and upregulated aggrecan levels, which is crucial for maintaining cartilage integrity (Saberianpour et al. 2022)
Atorvastatin	Mitigated inflammatory responses, alleviated pain, normalized bone metabolism, and enhanced bone mineral density across various skeletal sites in osteoporotic patients (Zhang et al. 2019) Alleviated joint stiffness, reduced histopathological abnormalities, suppressed elevated levels of MMP-13 along with IL-1β, and restored glutathione concentrations in osteoarthritis models (Gaballah et al. 2022) Improved arthritis symptoms and protected cartilage from degradation by reducing oxidative stress in MIA-induced osteoarthritis models (Pathak et al. 2015) Preserved cartilage integrity by preventing IL-1β-induced damage in in vitro studies, thus highlighting its chondroprotective properties (Assirelli et al. 2014)

IL, interleukin; MMP, matrix metalloproteinase.

### HMG-CoA reductase inhibitors and cancer

It has been assumed that the anti-cancer potentials of statins are attributed to their classical effect on cholesterol synthesis, as well as their pleiotropic effects on apoptosis, autophagy, angiogenesis, and metastasis. Several investigations have been made on the anti-cancer activities of statins, where it has been observed that statins inhibit tumor progression and provide a prolonged survival rate (Wang et al. 2016; Iarrobino et al. 2018; Chimento et al. 2019). In addition, it is believed that the anti-cancer effects of statins are due to the direct inhibition of HMG-CoA reductase, because the later effect ultimately restricts protein prenylation, GTPases, Ras, and RhoA (Göbel et al. 2020; Jiang et al. 2021).

It has been revealed that statins could promote apoptosis of different cancer cell types, including prostatic, breast, lung, liver, as well as colorectal cancers (Zaleska et al. 2018; Guo et al. 2022). Dehnavi and his colleagues (Dehnavi et al. 2021) concluded that statins, by means of activating AMPK, could limit cancer cell proliferation and promote apoptotic cell death. In the same context, (Okubo et al. 2021) have found that simvastatin promotes apoptosis in bladder cancer cells by means of histone acetylation via AMPK activation. Additionally, simvastatin has been found to foster cell cycle arrest in hepatocellular cancer through AMPK activation, with subsequent p21/27 elevation and STAT3 suppression (Wang et al. 2017b).

Chou and his coworkers (Chou et al. 2019) have demonstrated that simvastatin promotes the degradation of mutant p53 proteins in lung cancer and accelerates caspase-dependent apoptosis. Furthermore, in patient-derived xenograft (PDX) models with elevated geranylgeranyl diphosphate synthase 1 (GGPS1) levels, statins have been found to suppress GGPS1/Ras-related protein Rab-7a (RAB7A) autophagy axis, which in turn potentiates oxidative stress and apoptosis in lung carcinoma (Guo et al. 2022). Meanwhile, in colorectal cancer, simvastatin has been revealed to promote apoptosis through decreasing Bcl-2 as well as the cellular FADD-like IL-1β-converting enzyme inhibitory protein (cFLIP), and increasing caspase-3 (Cho et al. 2008b).

Statins reduce the viability of cancer cells by triggering autophagy in a variety of cancers (Ashrafizadeh et al. 2020; Mengual et al. 2022). On the contrary, statins can also cause apoptosis by blocking autophagy (Chou et al. 2019; Guo et al. 2022). Interestingly, statin-mediated autophagy has been linked to the control of a number of signaling pathways, such as AMPK/p21, AMPK/mammalian target of rapamycin (mTOR), and p53 (Yang and Chen 2011; Zhang et al. 2012). In a study performed on breast cancer cell lines, it was found that fluvastatin activates autophagy via AMPK/mTOR pathway, which inhibits cancer cell growth (Elimam et al. 2020). Similar outcomes have been reported with lovastatin in mesothelioma (Asakura et al. 2011). In the study of (Yang et al. 2010), it has been concluded that atorvastatin stimulates AMPK/p21 leading to autophagy induction in hepatocellular carcinoma and colorectal cancer. Intriguingly, comparable results have been reported in vivo with fluvastatin (Yang et al. 2017).

### HMG-CoA reductase inhibitors and eye disorders

#### Overview

Driven by their anti-inflammatory and antioxidative credentials, as well as their ability to augment ocular blood flow, statins can have a place in different ocular disorders. Interestingly enough, statins can also inhibit new blood vessels formation, which may assist in managing disorders associated with aberrant vascular proliferation. Moreover, statins may save retinal ganglion cells from apoptosis and enhance neuronal survival.

#### Diabetic retinopathy

Diabetic retinopathy, a primary contributor to adult blindness, is a well-known microvascular complication of diabetes. It is characterized by injury to retinal blood vessels, which may result in visual loss and blindness. Diabetic retinopathy has two primary stages: the non-proliferative diabetic retinopathy (NPDR) phase and the proliferative diabetic retinopathy (PDR) phase. During NPDR, retinal blood vessels weaken, resulting in the formation of small knots, known as microaneurysms. These microaneurysms may leak fluids, resulting in retinal enlargement (macular edema). In the advanced stage, i.e. PDR, the retina initiates the formation of new blood vessels via a process known as neovascularization. These newly formed arteries are delicate and are prone to hemorrhage, which can result in significant visual impairment (Murakami et al. 2021; Lymperopoulou et al. 2023; Tomkins-Netzer et al. 2024).

Research indicates that statins may have a preventive effect against diabetic retinopathy. A comprehensive review conducted by Lymperopoulou et al. (2023) emphasized the possible advantages of statins in mitigating the course of diabetic retinopathy. Tomkins-Netzer et al. (2024) as well underscored the implication of statins in the management of diabetic retinopathy, especially in patients with concomitant hypercholesterolemia. Murakami et al. (2021) showed that rigorous statin treatment may ameliorate the severity of diabetic retinopathy in hypercholesterolemic individuals. The possible amendments endured by statins encompass endothelial function enhancement and inflammation mitigation. Besides, statins can improve blood flow in retinal vessels and impede the development of aberrant blood vessels, thus reducing microvascular complications and PDR, respectively (Murakami et al. 2021; Lymperopoulou et al. 2023; Tomkins-Netzer et al. 2024).

#### Glaucoma

Glaucoma, a collection of visual disorders that impair the optic nerve, is often linked to elevated intraocular pressure (IOP). Because the optic nerve transmits visual information from the eye to the brain, injury to this nerve may result in permanent blindness. Various forms of glaucoma exist, including open-angle glaucoma and angle-closure glaucoma. The predominant form, i.e. open-angle glaucoma, is characterized by progressive obstruction of the ocular drainage pathways, resulting in gradual and asymptomatic elevation of IOP. Meanwhile, the angle-closure glaucoma condition arises when the drainage angle between the iris and cornea is obstructed, resulting in rapid and painful elevation in IOP.

Although data supporting statin usage in glaucoma is scarce, several trials highlighted possible advantages. Ooi et al. (2019) examined the prospective utility of statins in glaucoma, given their neuroprotective properties. Again, possible modes of action in glaucoma encompass neuroprotection and ocular blood flow enhancement. Statins may also save retinal ganglions from apoptosis, hence maintaining optic nerve functionality. Furthermore, through augmenting blood circulation to the optic nerve, ischemic injury can be mitigated by statins (Ooi et al. 2019).

## Pharmacokinetic differences and frequently occurring drug interactions among different statin members

Statins are divided into two main categories based on their solubility: lipophilic (atorvastatin, fluvastatin, lovastatin, simvastatin) and hydrophilic (pitavastatin, pravastatin, rosuvastatin). Lipophilic statins can passively diffuse into cells, whereas hydrophilic statins require transporter proteins to cross cell membranes. Notably, lovastatin and simvastatin are administered as inactive lactone prodrugs that must be converted into their active forms, while other statins are given as active acids (Fong 2014).

Absorption of statins occurs rapidly, with peak plasma concentrations typically being reached within 4 h. However, due to extensive first-pass metabolism in the intestinal wall and liver, their systemic bioavailability varies significantly. Simvastatin and lovastatin have very low bioavailability (< 5%), while atorvastatin and rosuvastatin have moderate levels (12% and 20%, respectively). Pitavastatin, in contrast, has high bioavailability (> 60%). Plasma protein binding is the highest for lipophilic statins (~ 95%), whereas rosuvastatin and pravastatin exhibit lower binding (90% and 50%, respectively). Since statins are highly protein-bound, displacement by other drugs is unlikely to cause clinically significant interactions (Palleria et al. 2020).

The elimination half-lives of statins also differ widely. Fluvastatin, lovastatin, pravastatin, and simvastatin have short half-lives (< 5 h), pitavastatin has an intermediate half-life (~ 13 h), and atorvastatin and rosuvastatin have prolonged half-lives (15–30 h). The metabolism of lipophilic statins is primarily mediated by cytochrome P450 (CYP) enzymes. Atorvastatin, lovastatin, and simvastatin are mainly metabolized by CYP3A4/5 enzymes in both the intestine and liver, with simvastatin also undergoing minor metabolism via CYP2C8. Fluvastatin demonstrates a unique metabolic profile, with 50–80% of its metabolism mediated by CYP2C9 and only minimal involvement of CYP3A4 and CYP2C8. In contrast, hydrophilic statins like rosuvastatin and pravastatin are largely excreted unchanged, with only minor metabolism occurring through various CYP enzymes. Most statin metabolites remain pharmacologically active, except those of fluvastatin and pravastatin (Williams and Feely 2002; Bellosta and Corsini 2018).

The primary elimination route for most statins is through biliary excretion following hepatic metabolism. This pathway makes hepatic impairment a significant risk factor for statin-induced myopathy. However, pravastatin and rosuvastatin exhibit dual elimination pathways, being excreted through both renal and hepatic mechanisms largely in their unmetabolized form (Schachter 2005). In addition, statins are influenced by various efflux and uptake transporters in the liver, intestines, and kidneys, which play a key role in their pharmacokinetics and contribute to potential drug-drug interactions (DDIs). Among these, the organic anion-transporting polypeptide 1B1 (OATP1B1) facilitates the hepatic uptake of all statins. Additional transporters involved in statin absorption and elimination include OATP1B3, OATP2B1, and multidrug resistance-associated proteins. Notably, P-glycoprotein (P-gp) mediates the efflux of several statins, such as simvastatin, fluvastatin, atorvastatin, and rosuvastatin, while breast cancer resistance protein also contributes to their transport (Neuvonen et al. 2006; Palleria et al. 2020).

## High-risk statin-drug interactions

Below is a brief listing for the alarming DDIs of statins (Maggo et al. 2011; Kellick et al. 2014; Cid-Conde and López-Castro, 2020; Palleria et al. 2020).

### CYP3A4 inhibitors (most relevant for simvastatin, lovastatin, atorvastatin)

These drugs include: macrolide antibiotics (clarithromycin, erythromycin), antifungals (itraconazole, ketoconazole, fluconazole, posaconazole), calcineurin inhibitors (cyclosporine), HIV protease inhibitors (ritonavir, atazanavir, darunavir), calcium channel blockers (verapamil, diltiazem), and grapefruit juice, especially in large quantities.

### CYP2C9 inhibitors (affect fluvastatin, rosuvastatin to a lesser extent)

Some examples for these drugs are fluconazole and amiodarone (also weak CYP3A4 inhibition).

### OATP1B1 inhibitors (affect all statins, especially simvastatin, atorvastatin, rosuvastatin, pitavastatin)

Examples for these medications are cyclosporine, gemfibrozil, protease inhibitors (e.g., ritonavir).

## Statin-associated myopathy

Statin-associated myopathy can be categorized into four distinct clinical presentations based on severity and underlying mechanisms: rhabdomyolysis, myalgia/mild hyperCKemia, self-limited toxic statin myopathy, and immune-mediated necrotizing myopathy (IMNM) associated with anti-HMG-CoA reductase antibodies (Mohassel and Mammen 2013; Alfirevic et al. 2014).

### Rhabdomyolysis

Rhabdomyolysis is the most severe but rare form of statin-induced muscle injury, occurring in fewer than 1 per 100,000 treated patients annually (Law and Rudnicka 2006). It is characterized by markedly elevated creatine kinase (CK) levels (> 100 times the upper limit of normal), along with myoglobinuria and acute kidney injury due to myoglobin-induced tubular necrosis (Bosch et al. 2009).

### Myalgia and mild HyperCKemia

This milder form of statin-related muscle toxicity is common in clinical practice, with myalgia (muscle pain) being the most frequent symptom and a leading cause of statin discontinuation. While clinical trials report a myalgia incidence of ~ 9.4% (similar to placebo) (Taylor et al. 2013), observational studies suggest a higher prevalence (~ 20%), possibly due to the exclusion of high-risk patients in trials (Bruckert et al. 2005; Parker et al. 2013; Stroes et al. 2015).

Mild CK elevations (< 5 times the upper limit, typically < 1000 IU/L) may accompany myalgia but do not always necessitate statin withdrawal. Interestingly, symptoms may persist after stopping statins in some patients, while others improve despite continued therapy (Hansen et al. 2005; Armour and Zhou 2013).

### Self-limited toxic statin myopathy

A non-immune-mediated toxic myopathy can develop in some patients, presenting with progressive muscle weakness (sometimes severe enough to impair daily activities) and CK elevations between 10–100 times the upper limit (2000–20,000 IU/L). Myalgia is also common, and symptoms typically resolve after statin discontinuation (Selva-O'Callaghan et al. 2018).

### IMNM with anti-HMG-CoA reductase antibodies

IMNM is a rare but serious autoimmune myopathy linked to statin use, with an estimated incidence of 2–3 cases per 100,000 statin-exposed patients (Mammen 2016). Key features include: progressive proximal muscle weakness, CK levels 10–100 times above normal (2000–20,000 IU/L), myopathic electromyography findings and muscle biopsy showing necrosis, regeneration, and minimal inflammation (mainly macrophages) (Christopher-Stine et al. 2010; Nazir et al. 2017). IMNM has a genetic predisposition, with HLA-DRB1*11:01 in adults and DRB1*07:01 in children being common risk alleles (Mammen et al. 2012; Kishi et al. 2017).

## Quantitative differences, relative risks and relative benefits of different statins

Statin medications are a cornerstone of CV risk reduction, with well-documented efficacy across multiple large randomized controlled trials. However, they differ in potency, safety profiles, and clinical outcomes. Key studies have compared different statins in terms of clinical outcomes and adverse effects, providing insights into their relative benefits and risks (Moride et al. 2008; Khatiwada and Hong 2024a).

### Comparative efficacy in major clinical trials

The Heart Protection Study (HPS), which included 20,536 high-risk individuals over 5.3 years, showed that simvastatin (40 mg/day) significantly reduced major vascular events compared to placebo, with sustained benefits over an 11-year follow-up (Heart Protection Study Collaborative 2011). Similarly, the ASCOT-LLA trial (Anglo-Scandinavian Cardiac Outcomes Trial—Lipid-Lowering Arm) evaluated atorvastatin (10 mg/day) in 10,305 hypertensive patients and found a notable reduction in CV events after 3.3 years (Sever et al. 2004).

For patients without prior CVD but with elevated inflammation markers (high-sensitivity CRP), the JUPITER trial (Justification for the Use of Statins in Prevention: An Intervention Trial Evaluating Rosuvastatin) demonstrated that rosuvastatin (20 mg/day) significantly lowered CV risk in 17,802 participants over two years as compared to placebo (Thompson et al. 2016). In contrast, the PROVE-IT TIMI 22 trial (Pravastatin or Atorvastatin Evaluation and Infection Therapy-Thrombolysis in Myocardial Infarction 22) compared high-intensity atorvastatin (80 mg/day) with moderate-intensity pravastatin (40 mg/day) in 4,162 patients with recent acute coronary syndrome (ACS). The results favored high-dose atorvastatin, supporting aggressive lipid-lowering post-ACS (Cannon et al. 2004). Further reinforcing the dose-dependent benefits of statins, the TNT trial (Treating to New Targets) followed 10,001 patients with stable coronary disease, finding that atorvastatin 80 mg/day was more effective than 10 mg/day in reducing CV events over 4.9 years (Mora et al. 2012).

### Comparative safety profiles

When comparing safety profiles, statins differ in their risk of adverse effects. Myalgia risk varies among statins, with simvastatin showing lower odds of muscle pain compared to atorvastatin (OR 0.56, 95% CI 0.42–0.75).

Liver enzyme elevations were more frequent with atorvastatin and fluvastatin, whereas pravastatin, rosuvastatin, and simvastatin had significantly lower risks. Specifically, pravastatin was associated with the lowest odds of transaminase increases (OR 0.27 *vs.* atorvastatin). Fluvastatin had the highest risk, with 5.2 times greater odds than placebo and significantly more than other statins.

Regarding CK elevations, pitavastatin showed the highest risk (OR 3.63 *vs.* control), while fluvastatin had the lowest, comparable only to lovastatin.

In summary, statins vary in potency, CV benefits, and adverse effect profiles. High-intensity statins (atorvastatin, rosuvastatin) are preferred for secondary prevention, while moderate-intensity statins (simvastatin, pravastatin, pitavastatin) may be better tolerated in lower-risk populations. Individualized treatment should consider efficacy, safety, and patient-specific factors (e.g., drug interactions, comorbidities) (Alsheikh-Ali et al. 2007; Silva et al. 2007; Naci et al. 2013a).

## Overall benefit-risk assessments

Statins consistently demonstrated superior effectiveness over control treatments in lowering the risk of all-cause mortality (OR 0.87, 95% CI 0.82–0.92) and major coronary events (OR 0.69, 95% CI 0.64–0.75) across various populations. Notably, atorvastatin (OR 0.66, 95% CI 0.48–0.94) and fluvastatin (OR 0.59, 95% CI 0.36–0.95) achieved greater reductions in major coronary events compared to rosuvastatin at equivalent doses. In patients with established CVD, statin therapy significantly reduces mortality (OR 0.82, 95% CI 0.75–0.90) and coronary events (OR 0.69, 95% CI 0.62–0.77). Among these individuals, atorvastatin demonstrates superior coronary event reduction relative to **pravastatin** (OR 0.65, 95% CI 0.43–0.99) and simvastatin (OR 0.68, 95% CI 0.38–0.98).

For primary prevention settings, statins significantly lowered mortality risk (OR 0.91, 95% CI 0.83–0.99) and major coronary events (OR 0.69, 95% CI 0.61–0.79), although no significant differences were observed between individual statins. Based on the combined outcomes, atorvastatin (80%), fluvastatin (79%), and simvastatin (62%) had the highest likelihood of being the most effective treatments overall. High-dose regimens of atorvastatin and fluvastatin were particularly notable for producing the most significant differences in event reduction when compared to other statins. No significant heterogeneity or inconsistency was identified across studies (Naci et al. 2013c).

Regarding cerebrovascular outcomes, statin use significantly decreased the occurrence of major cerebrovascular events (OR 0.82, 95% CI 0.77–0.87) across all populations, with no statistically significant differences between individual agents. Both patients with and without established CVD benefited from this reduction (OR 0.83, 95% CI 0.75–0.91 and OR 0.80, 95% CI 0.71–0.91, respectively). Among individual statins, atorvastatin (OR 0.74, 95% CI 0.63–0.85), pravastatin (OR 0.86, 95% CI 0.76–0.97), and simvastatin (OR 0.75, 95% CI 0.62–0.88) demonstrate significant reductions in cerebrovascular events versus control. Statin therapy also reduced the risk of non-fatal stroke (OR 0.77, 95% CI 0.71–0.85), while no significant effect was observed for fatal stroke (OR 0.96, 95% CI 0.80–1.15). These findings remain consistent across dosage levels, with no evidence of heterogeneity or inconsistency (Naci et al. 2013b).

In conclusion, the data robustly support the class-wide benefit of statins in reducing coronary and cerebrovascular events, with atorvastatin, fluvastatin, and simvastatin emerging as particularly effective options. The favourable risk–benefit profile reinforces their use in both primary and secondary prevention.

## Natural products as HMG-CoA reductase inhibitors

**Fig. 1:**
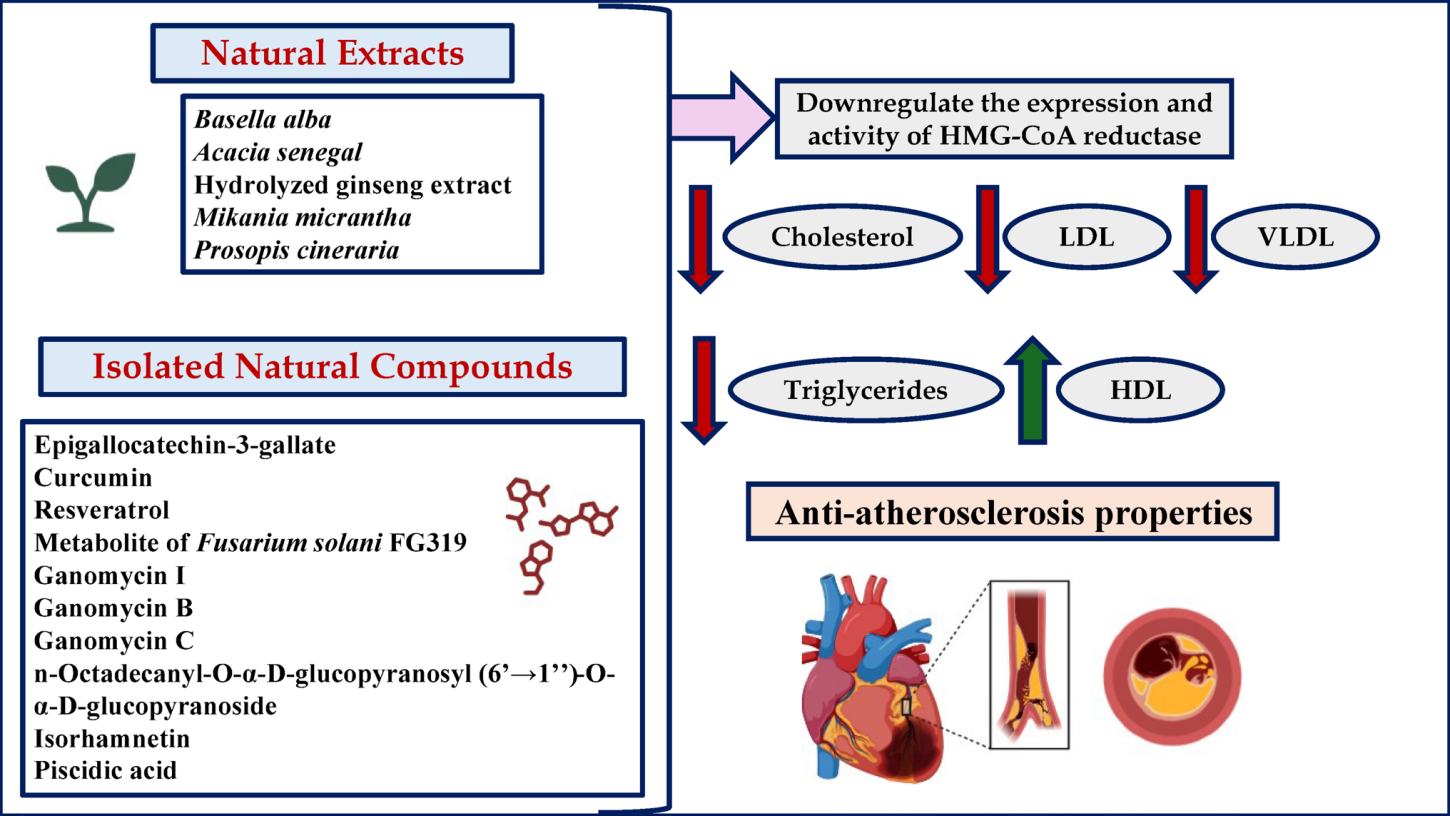
Schematic representation for the role of different natural products in the inhibition of HMG-CoA reductase enzyme. Abbreviations: HDL, high density lipoprotein; HMG-CoA, hydroxymethylglutaryl-CoA; LDL, low density lipoprotein; VLDL, very low-density lipoprotein

**Fig. 2:**
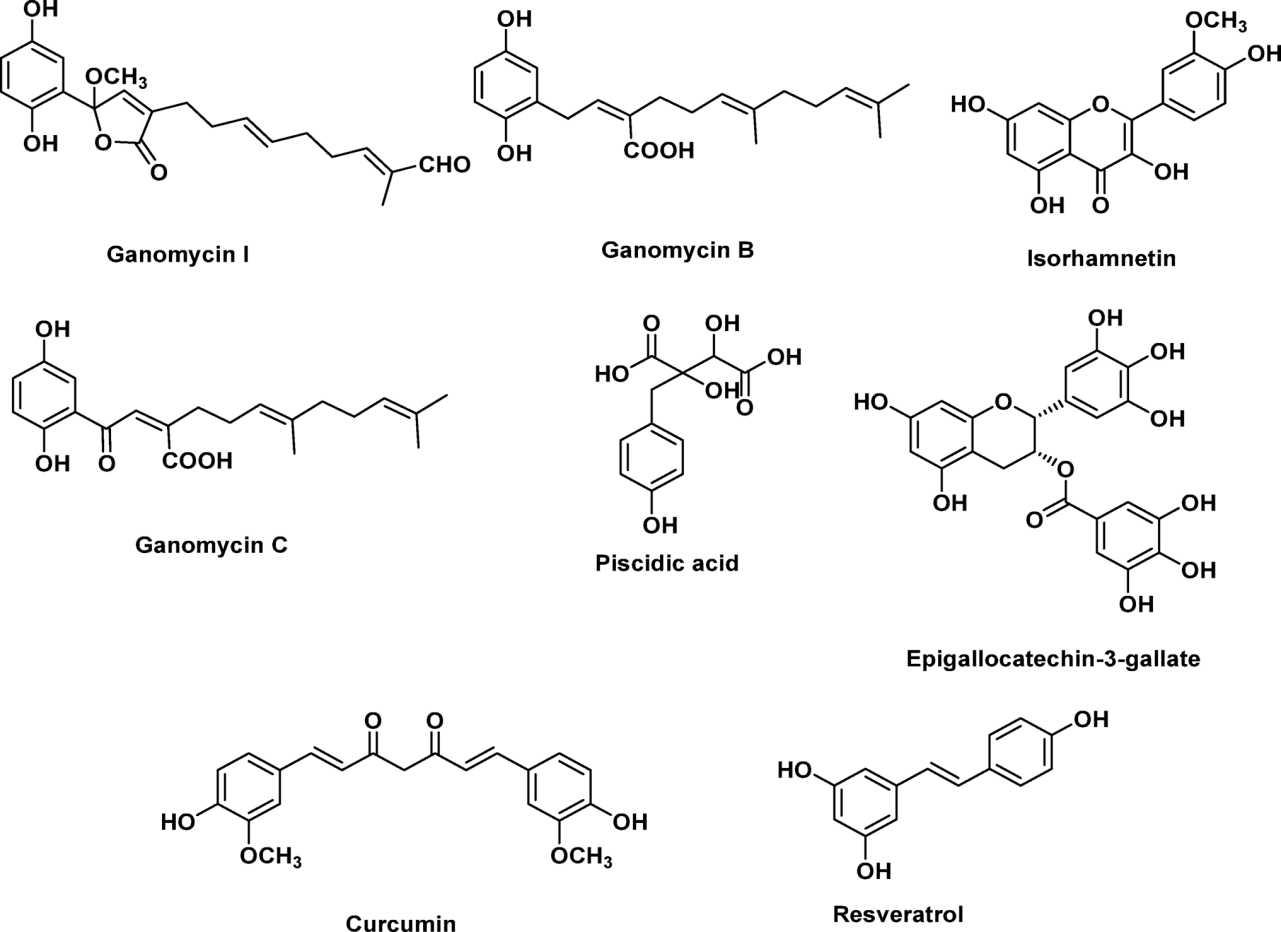
Chemical structures of different natural products with a potential inhibitory effect on HMG-CoA reductase. Abbreviations: HMG-CoA, hydroxymethylglutaryl-CoA

Many natural products have a long history of biological activity and operate as precursor compounds for the formulation of new pharmaceuticals (Aly et al. 2023; Abdelazim et al. 2024). Natural products exhibit a diversity of chemical structures as flavonoids, alkaloids, phenolic acids, terpenoids…etc. (Aly et al. 2020, 2024). This diversity is crucial for discovering new therapeutic agents that can effectively target complex biological systems (Elebeedy et al. 2023; Cusumano et al. 2024; Zengin et al. 2024). Natural compounds, especially those derived from medicinal plants and fungi, exhibit potential effects as HMG-CoA reductase inhibitors (Mahdavi et al. 2020). Their capacity to reduce cholesterol levels while mitigating negative effects renders them appealing alternatives to conventional statin therapy. Additional investigation into these compounds may improve the knowledge of their mechanisms and effectiveness, facilitating the development of novel therapies for hypercholesterolemia and associated CVD (Figs. 1 and 2).

The *Basella alba* (Basellaceae) leaf extract demonstrated a significant HMG-CoA reductase inhibitory effect, achieving about 74% inhibition as compared to simvastatin, which showed enzyme inhibition of 85.1%. This positions *Basella alba* as a promising candidate for reducing cholesterol biosynthesis, similar to the action of statins but potentially with fewer side effects. Phytochemical analysis using gas chromatography and high-performance liquid chromatography identified several bioactive compounds in the extract, including oleic acid, luteolin, apigenin, naringin, ascorbic acid and α-tocopherol. These components are known for their antihypercholesterolemic properties, supporting the extract’s therapeutic potential (Baskaran et al. 2015).

The impact of the aqueous seed extract of *Acacia senegal* (L.) Willd on dyslipidaemia and atherosclerotic plaque development was examined, emphasizing its capacity to inhibit HMG-CoA reductase enzyme. The extract demonstrated notable 74.1% inhibition of HMG-CoA reductase activity in *vitro*, in contrast to pravastatin-mediated 91.4% inhibition, suggesting its potential as a natural beneficial substance for cholesterol management. Hypercholesterolemic rabbits with a 400 mg/kg *Acacia senegal* seed extract exhibited significant enhancements in multiple biomarkers related to dyslipidaemia, including the Atherogenic index, Castelli risk index (I & II), and Atherogenic coefficient. Additionally, the treatment led to notable reductions in both TC and TG levels, thus contributing to better lipid profiles. Furthermore, in silico analyses revealed that key phytoconstituents such as eicosanoic acid, linoleic acid, and flavan-3-ol interacted effectively with HMG-CoA reductase, supporting their potential as active compounds in the extract (Charan et al. 2022).

Another investigation examined the impact of GINST, a hydrolyzed ginseng extract, on cholesterol synthesis in HepG2 cells, emphasizing its mechanism related to HMG-CoA reductase and AMPK. GINST substantially suppressed cholesterol synthesis in HepG2 cells, resulting in TC levels markedly diminished after 24 and 48 h of treatment (30 μg/mL, 86.64% of control; 90 μg/mL, 79.96%; 270 μg/mL, 73.28%). The inhibition was more significant than that attained with conventional inhibitors such as atorvastatin. The effects would be ascribed to the stimulation of AMPKα phosphorylation. The principal bioactive constituent extracted from GINST, IH-901 (compound K), a ginsenoside, exhibited notable cholesterol-lowering effects in HepG2 cells ranging from 75.51 to 84.41% of control upon using different concentrations (0.1, 0.3, 1 μg/mL), thereby substantiating the extract’s efficacy in addressing hypercholesterolemia (Han et al. 2017).

The impact of the ethyl acetate extract of *Mikania micrantha* on hypercholesterolemia and lipid peroxidation in rats subjected to a high-cholesterol diet was examined at dosages of 50, 100, and 200 mg/kg, emphasizing its capacity to inhibit HMG-CoA reductase and acyl-CoA: cholesterol acyltransferase 2 (ACAT2). The extract demonstrated considerable suppression of both HMG-CoA reductase and ACAT2 activities in comparison to simvastatin (10 mg/kg). Moreover, treatment with the extract led to substantial decreases in TC, TG, and LDL (Ibrahim et al. 2020).

Another study explored the effects of an ethanolic pod extract of *Prosopis cineraria* on hypercholesterolemia and atherosclerotic plaque regression, focusing on its ability to inhibit HMG-CoA reductase. The extract demonstrated substantial suppression of HMG-CoA reductase activity, achieving 67.1% inhibition in vitro*.* This is comparable to the standard drug pravastatin, which achieved 97.3% inhibition, which pinpoints the extract’s potential as a natural cholesterol-lowering agent (Ram et al. 2020).

Metabolite of *Fusarium solani* FG319 (MFS), a purified extract derived from the fermentation of *F. solani* FG319, exhibited a superior inhibitory impact on HMG-CoA reductase compared to the positive control, lovastatin. The IC_50_ of MFS against HMG-CoA reductase was determined to be 3.672 μg/mL in vitro*—*a value which is markedly lower than that of lovastatin at 77.830 μg/mL. The in vitro inhibitory impact of MFS on HMG-CoA reductase was significantly greater than that of lovastatin (Yu et al. 2015).

Different meroterpenoids have been isolated from the fruiting bodies of the fungus *Ganoderma leucocontextum* (Ganodermataceae, Polyporales). Ganomycin I, ganomycin B, and ganomycin C exhibited superior inhibitory activity against HMG-CoA reductase compared to the positive control atorvastatin, with IC_50_ values of 12.3 ± 1.7, 29.3 ± 2.5, and 45.2 ± 7.1 µM, respectively (Atorvastatin IC_50_ = 32.1 ± 7.7 µM). The in vivo investigation showed that following 3 weeks of therapy with ganomycin I at a dosage of 1.5 mg/kg/day, the serum concentrations of non-esterified fatty acids (NEFA), TG, TC, and LDL were considerably reduced in insulin-resistant male KK-A^y^ mice compared to the KK-A^y^ diabetic animals. The study determined that ganomycin I demonstrated significant hypoglycemic, hypolipidemic, and insulin-sensitizing effects in KK-A^y^ mice relative to rosiglitazone. Moreover, the inhibitory effects of HMG-CoA reductase may enhance lipid profiles in diabetic individuals, hence promoting CV health (Wang et al. 2017a).

Another investigation successfully extracted a bioactive molecule designated *n*-octadecanyl-*O*-α-D-glucopyranosyl (6’ → 1’’)-O-α-D-glucopyranoside (termed F18) from the methanolic extract of *Ficus virens* bark. This chemical demonstrated substantial inhibition of HMG-CoA reductase, with an IC_50_ value of 84 ± 2.8 ng/mL, in contrast to pravastatin, which had an IC_50_ value of 70 nM. The in vivo study revealed that administering *Ficus virens* bark methanolic (FVBM) extract (50/100 mg) and atorvastatin (1 mg) to Triton WR-1339 hyperlipidaemic rats significantly ameliorated the disrupted plasma lipid and lipoprotein levels, as well as hepatic HMG-CoA reductase activity. Of note, these effects were comparable to that of the standard drug, atorvastatin (Iqbal et al. 2015).

Phenolic compounds, such as isorhamnetin and piscidic acid derived from *Opuntia ficus-indica*, markedly decreased cholesterol permeability across a Caco-2 cell monolayer by 38%. This suggests their potential in limiting cholesterol absorption in the intestines. Isorhamnetin derivatives exhibited a notable inhibitory effect on HMG-CoA reductase, with an IC_50_ value of 20.3 μg/mL, while piscidic acid alone showed an IC_50_ of 149.6 μg/mL. This indicates that isorhamnetin derivatives are more effective inhibitors of this enzyme, which plays a critical role in cholesterol biosynthesis (Ressaissi et al. 2017).

A study conducted by Cocci et al. (2014) examined the effects of epigallocatechin-3-gallate (EGCG), a significant polyphenol presents in green tea, on goldfish. The treatment of EGCG led to a notable decrease in HMG-CoA reductase mRNA levels in the liver, indicating that EGCG may influence lipid metabolism in goldfish (Cocci et al. 2014).

Another investigation examined the preventive effects of prolonged curcumin treatment against atherosclerosis. It suppressed the transcription of HMG-CoA reductase and resulted in a marked enhancement of early atherosclerotic lesions and fat accumulation in the aortic arch, akin to the results seen with lovastatin. Furthermore, curcumin reduced plasma levels of TC, TGs, LDL, and apolipoprotein B (Apo B). Conversely, curcumin elevated HDL cholesterol levels and hepatic Apo A-I expression (Shin et al. 2011).

Resveratrol exhibited antioxidant and hypolipidemic actions that enhance its antiatherosclerotic capabilities. Real-time PCR analysis revealed that HMG-CoA reductase mRNA expression was markedly diminished in the resveratrol-fed group compared to controls (Cho et al. 2008a). A different investigation attempted to investigate the mechanisms via which resveratrol and simvastatin affect the mevalonate pathway, with specific emphasis on HMG-CoA reductase expression and activity. Resveratrol markedly decreased cholesterol production by suppressing HMG-CoA reductase expression and activity. Meanwhile, resveratrol, when coupled, augmented the inhibitory effects of simvastatin on HMG-CoA reductase activity and cholesterol production (Wong et al. 2011).

## Conclusion

Herbal medicines, particularly *Basella alba*, *Acacia senegal*, and ginseng, in addition to natural compounds as curcumin, resveratrol, isorhamnetin, and EGCG have demonstrated beneficial effects on the expression and activity of HMG-CoA reductase, an enzyme crucial for cholesterol biosynthesis. Research indicates that these herbal substances can effectively decrease the levels of HMG-CoA reductase, thereby presenting a promising avenue for the prevention of NCDs due to their accessibility and cost-effectiveness as natural alternatives to the synthetic statin drugs. Despite these promising findings, further clinical trials are essential to confirm the efficacy of these nutraceuticals. Such studies should aim to determine optimal dosages and treatment durations necessary to achieve significant reductions in HMG-CoA reductase activity in human subjects. Additionally, exploring the potential therapeutic effects of these herbal medicines could provide valuable insights into their role as complementary treatments alongside conventional therapies for managing cholesterol levels and preventing NCDs.

## Data Availability

No datasets were generated or analysed during the current study.
